# BMP2 promotes lung adenocarcinoma metastasis through BMP receptor 2-mediated SMAD1/5 activation

**DOI:** 10.1038/s41598-022-20788-2

**Published:** 2022-09-29

**Authors:** Cheng-Kuei Wu, Man-Ting Wei, Hung-Chang Wu, Cheng-Lin Wu, Cheng-Ju Wu, Hungjiun Liaw, Wen-Pin Su

**Affiliations:** 1 Institute of Clinical Medicine, College of Medicine, National Cheng Kung University, No. 35, Xiao-Tong Road, Tainan, 704 Taiwan; 2 Division of Hematology and Oncology, Department of Internal Medicine, Chi-Mei Medical Center, Tainan, Taiwan; 3 Department of Pharmacy, Chia-Nan University of Pharmacy and Science, Tainan, Taiwan; 4 Department of Pathology, National Cheng Kung University Hospital, College of Medicine, National Cheng Kung University, Tainan, Taiwan; 5 Departments of Oncology and Internal Medicine, National Cheng Kung University Hospital, College of Medicine, National Cheng Kung University, Tainan, Taiwan; 6 Department of Life Sciences, National Cheng Kung University, No. 1 University Road, Tainan City, 701 Taiwan

**Keywords:** Lung cancer, Non-small-cell lung cancer, Cancer, Oncology

## Abstract

Bone morphogenetic protein 2 (BMP2) is highly overexpressed in human non-small cell lung cancer (NSCLC) and correlates with tumor stage and metastatic burden. Although several lines of evidence suggest that BMP2 promotes cell migration and invasiveness in vitro, the in vivo role of BMP2 in the metastasis of lung adenocarcinoma cells remains less well understood. Here, we revealed that BMP2 is highly overexpressed in lung adenocarcinoma patients with lymph node metastasis compared with patients without lymph node metastasis. Using an in vivo orthotopic mouse model, we clearly demonstrated that BMP2 promotes lung adenocarcinoma metastasis. The depletion of BMP2 or its receptor BMPR2 significantly reduced cell migration and invasiveness. We further identified that BMP2/BMPR2-mediated cell migration involves the activation of the SMAD1/5/8 signaling pathway, independent of the KRAS signaling pathway. Significantly, the depletion of SMAD1/5/8 or the inhibition of SMAD1/5/8 by LDN193189 inhibitor significantly reduced cell migration. These findings show that BMP2 promotes NSCLC metastasis, indicating that targeting the BMP2 signaling pathway may represent a potential therapeutic strategy for treating patients with metastatic NSCLC.

## Introduction

Lung cancer is the most common cancer type and the leading cause of cancer deaths in both men and women worldwide. From 2010 to 2016, the 5‐year relative survival rate for lung cancer was only 21% with the majority of patient deaths caused by complications from metastasis^1^. Lung cancer originates from respiratory epithelial cells and can be separated into two major categories: non-small cell lung cancer (NSCLC), which represents approximately 85% of all lung cancer cases, and small cell lung cancer (SCLC), which represents the remaining 15% of cases^2^. NSCLC is characterized by widespread metastasis at an early stage. Therefore, understanding the mechanisms that drive lung adenocarcinoma metastasis represents an urgent clinical need.

Bone morphogenetic proteins (BMPs) are growth factors that belong to the transforming growth factor β (TGFβ) superfamily, and at least 20 isoforms have been identified in humans to date^3,4^. BMPs participate in various cellular activities, including proliferation, differentiation, migration, invasion, and apoptosis^3^. BMP synthesis starts with a precursor protein consisting of an N-terminal domain that regulates secretion and the C-terminal mature domain^5^. Following either homo- or heterodimerization, the precursor protein is cleaved, releasing the mature C-terminal peptide. The mature BMP peptides bind to type I and type II serine/threonine kinase receptors, triggering SMAD1/5/8 phosphorylation^3^. The phosphorylated SMAD1/5/8 binds with SMAD4, translocates to the nucleus, and activates transcription in conjunction with specific transcription factors^3–7^.

The highly complex BMP signaling pathway can be regulated by various combinations of BMP molecules, antagonists, and type I and type II kinase receptors, resulting in different BMP signaling outcomes that depend on tumor types, BMP molecules involved, and genetic background, which may be responsible for the conflicting results reported in past studies. For example, some studies have revealed that BMP signaling functions as a tumorigenesis barrier. Mutations in BMP receptor 1A (BMPR1A) and SMAD4 have been identified in patients with juvenile polyposis (JP), who are also at high risk for colorectal cancer development, suggesting that BMP may serve as an important barrier preventing intestinal tumor initiation^4,8,9^. Consistently, BMP4 induces SMAD1/5/8 phosphorylation, which profoundly inhibits tumor growth^10^. The administration of the BMP antagonist Coco inhibits SMAD phosphorylation and induces metastasis in breast cancer cells^10^. Similarly, the BMP antagonist noggin (NOG), inhibits BMP signaling and promotes breast cancer metastasis^11^. BMP7 limits cancer stem-like cell (CSC) growth, and the BMP7 depletion promotes CSC proliferation both in vitro and *in vivo*^12^. BMP7 expression levels are significantly correlated with recurrence-free survival^12^. By contrast, other studies have demonstrated that BMP signaling promotes cell proliferation and metastasis^13–21^. Consistently, expression of a dominant negative BMPR2 in vitro decreases breast cancer cell proliferation^22^. BMP2 is highly overexpressed in human NSCLC tissue compared with normal lung tissue or benign lung tumors^13–18,23^. Moreover, serum BMP2 levels positively correlated with tumor stage and metastatic burden, suggesting that BMP2 might serve as a prognostic marker for survival in NSCLC patients^15,16,20^. In vivo xenograft mouse models reveal that BMP2 signaling pathway enhances bone metastasis of mouse Lewis lung carcinoma^24^ and breast cancer cells^25^. In addition, BMP2 induces breast cancer stemness and lung metastasis in the mouse model where breast cancer cells are injected via caudal vein^26^. The inhibition of BMP2 signaling by LDN193189 or depletion of BMP2 by microRNAs are cytotoxicity in lung cancer cell line A549, but not immortal non-tumorigenic BEAS-2B bronchial epithelial cells^27^, suggesting that targeting BMP2 signaling may serve as a therapeutic strategy for treating lung cancers.

Due to conflicting results reported by past studies, we attempted to determine the effect of BMP signaling in lung cancer metastasis using metastatic lung cancer cell lines, CL1-5 and PC14PE6/AS2 (AS2), which were derived from less metastasis parental cell lines, CL1-0 and PC14PE6, respectively. We found that BMP2 and mesenchymal markers are highly overexpressed in metastatic cells. Additionally, using an in vivo orthotopic mouse model, we demonstrated that the BMP2-depleted lung cancer cells are less metastatic than BMP2-proficient control cells. Recombinant human BMP2 (rhBMP2) significantly increased the migration and invasion abilities of lung adenocarcinoma cells, and the BMPR inhibitor LDN193189 decreased migration and invasion abilities. The effects of BMP2 signaling appear to be mediated by SMAD 1/5/8 phosphorylation. These data suggest that targeting BMP2 signaling may serve as a therapeutic strategy for treating patients with metastatic NSCLC.

## Materials and methods

### Ethical approval

All procedures in the study involving human participants were carried out in accordance with the ethical standards of the institutional and/or national ethical committee and with the 1964 Helsinki Declaration and its later amendments or comparable ethical standards. All experiments were performed in accordance with relevant guidelines and regulations by the Institutional Review Boards, National Cheng Kung University Hospital (B-ER-105-392). The institutional review board of National Cheng Kung University Hospital waived the need of informed consent because this study was conducted using the medical records of anonymized patients. All of the experimental protocols involving mice were approved by the Institutional Animal Care and Use Committee of NCKU (IACUC NO: 106095). Supervision of animal facilities by a board-certified veterinarian further ensured compliance with all ethical protocols. We also confirm that the study is reported in accordance with ARRIVE guidelines (https://arriveguidelines.org).

### Cell culture

Human lung adenocarcinoma carcinoma cell lines, CL1-0, CL1-5, A549, and H1299 cells were cultured in RPMI 1640 medium (Gibco Invitrogen, USA) supplemented with 10% feral bovine serum (FBS, Gibco) and 1% penicillin/streptomycin (Gibco Invitrogen, USA). PC14PE6 and AS2 cells were culture in Minimum Essential Medium alpha (MEMα) medium (Gibco Invitrogen, USA) with 10% FBS, 1% penicillin/streptomycin, 1 mM sodium pyruvate (Gibco Invitrogen, USA), and 2% MEM vitamin solution (Gibco Invitrogen, USA). CL1-5 cells, which are derived from CL1-0 cells, have higher invasive ability than CL1-0 cells^28^. The PC14PE6 cell line was received from the same source as those used by Yeh et al.^29^. AS2 cell line was established from ascites generated by PC14PE6, as previously described^29^.

### Antibodies and reagents

The antibodies used in this study included those targeting β-actin (NB600-501, Novus), E-cadherin (#3195, Cell Signaling Technology for western blot; 20,874–1-AP, Proteintech for IHC staining), occludin (13,409–1-AP, Proteintech), N-cadherin (ab53519, Abcam), Vimentin (#550,513, BD), Slug (sc-166476, Santa Cruz Biotechnology), BMPR2 (ab78422, Abcam), BMP2 (A0231, ABclonal), SMAD5 (#9517, Cell Signaling Technology), SMAD1 (#6944, Cell Signaling Technology), SMAD4 (sc-7966, Santa Cruz Biotechnology), and phospho-SMAD1/5 (ser463/465) (#9516, Cell Signaling Technology). Reagents included rhBMP-2 (R&D system), puromycin (Sigma-Aldrich), and LDN193189 (Cayman).

### siRNA and shRNA gene knockdown

BMP2 shRNA plasmids (TRCN0000058195 and TRCN0000058196) were obtained from the RNAi Core Facility at Academia Sinica in Taiwan. The lentivirus was generated by transfecting HEK293T cells with shRNA plasmids and two packaging plasmids, pCMV-ΔR8.91 and pMD.G, using a transfection reagent (Lipofectamine 2000, Invitrogen). After 4 h of transfection, the cell culture medium was replaced with bovine serum albumin (BSA)-containing medium. Lentiviruses were harvested at 48 and 72 h after transfection.

To generate gene-depleted cells, the target cells were infected with shRNA-containing lentivirus, and cells were selected with 2 μg/ml puromycin. Alternatively, cells were transfected with siRNA (Dharmacon) using a transfection reagent (Lipofectamine 2000, Invitrogen). The siBMP2 sequences were CGACAGAACUCAGUGCUAU, CCAGGUUGGUGAAUCAGAA, CGCCUUAAGUCCAGCUGUA, and GAUGCAAGAUGCUUUAGGA. The siBMPR2 sequences were GAACGCAACCUGUCACAUA, GCAUGAGCCUUUACUGAGA, GAAACAAGUAGACAUGUAU, and GAAGGUGGCCGAACUAAUU.

### Quantitative polymerase chain reaction (qPCR)

RNA was extracted using an RNA Mini Kit (Zymo, USA), and cDNA was generated by using an iScript cDNA Synthesis Kit (BIO-RAD, USA). mRNA levels were determined by qPCR using the SYBR green supermix (Roche, Switzerland) and an ABI StepOne Plus™ Real-Time PCR System (Thermo Fisher Scientific, USA). The relative mRNA expression levels were normalized to the levels of β-actin. The detailed sequences of primers are listed as follows: BMP2 forward: GCCTTAAGTCCAGCTGTAAGAGAC. BMP2 reverse: GTACAGCATCGAGATAGCACTGAG. ACTB forward: AAAACAACAATGTGCAATACAAGT. ACTB reverse: CTTAGTTGCGTTACACCCTTTCTT.

### Western blotting

Cells were harvested in RIPA lysis buffer (50 mM Tris–HCl, pH 7.4; 150 mM NaCl; 0.25% deoxycholic acid; 1% NP-40; 1 mM EDTA) with protease inhibitor cocktail (MD Biol). Samples (15 μg of protein) were separated by SDS-PAGE. Proteins were transferred to PVDF membranes (Millipore, Bedford, MA) and blocked in 5% skim milk in TBST (20 mM Tris, pH 7.6; 150 mM NaCl; 0.1% Tween 20) for 1 h. The membranes were incubated with primary antibodies overnight at 4 °C. The next day, the membranes were incubated with secondary antibodies at room temperature for 1 h, and the blots were developed using ECL Plus Western Blotting Detection Reagents (Amersham, UK).

### Migration and invasion Transwell assay

Cell migration assays were performed in 8 μm-pore polycarbonate filter Transwells (Millipore, USA) without Matrigel (BD, USA) coating. Cell invasion assays were performed in 8 μm-pore polycarbonate filter Transwells with matrigel (BD, USA) coating. Cells were seeded into the upper chamber in serum-free medium, and the lower chamber was filled with medium containing 10% FBS. The cells were fixed in methanol for 10 min and stained with Liu's solution (TONYAR BIOTECH, Taiwan). The numbers of migrating and invading cells were observed under a microscopy Olympus IX71 (Olympus, Japan), and cell numbers were counted by ImageJ in multiple fields from each filter.

### Immunofluorescence and confocal microscopy

Cells were seeded onto four-well chamber slides, fixed with 4% paraformaldehyde, and permeabilized with 0.1% Triton X-100. The cells were blocked with 1% BSA and stained with antibodies targeting specific proteins, followed by fluorescent-conjugated secondary antibodies. 4',6-diamidino-2-phenylindole (DAPI) was used to stain DNA. Cells were observed by confocal microscope NikonC1si (Nikon, Japan).

### Clinical specimens

Human lung tumor specimens were obtained from National Cheng Kung University Hospital (Tainan, Taiwan) under a protocol approved. 94 samples derived from patients (pathological stage I-IIIa) were subdivided into different tumor-node-metastasis (TNM) stages based on the lymph node and metastasis status of each patient. Tumor samples from humans and mice were paraffin-embedded and cut 5 μm thick tissue sections. Sections were deparaffinized, rehydrated, incubated with target antibody, and stained by VECTASTAIN Elite ABC HRP Kit (Vector Laboratories, UK) and DAB Peroxidase (HRP) Substrate Kit (Vector Laboratories, UK), following to the manufacturer’s protocols. Stained sections were accepted by a light microscope (Olympus BX51, Japan). The significance of BMP2 levels in samples with or without lymph node metastasis was determined by chi-square test. The median for survival time was determined by the Kaplan–Meier method using the Log-rank test^30^. The significance of BMP2 levels based on age, gender, smoking, TNM stage, and lymph nodes metastases was determined by chi-square tests. Quantitative analysis of fluorescence intensity was done at the original magnification using ImageJ software (1.53 K) .

### Analysis of Kaplan–Meier curves of overall survival from public databases

The Kaplan–Meier curves of overall survival data with lung adenocarcinoma was analyzed from gene expression profiling interactive analysis database (http://gepia.cancer-pku.cn/index.html). These databases include TCGA and GTEx projects^31^.

### Tumor xenograft animal model

NOD-SCID mice (6–8 weeks, male) were purchased from the National Laboratory Animal Center (Tainan, Taiwan). The mice were anesthetized by intraperitoneal injection of Zoletil® 100 (Virbac, France). Stable shRNA-expressing cells (2 × 10^5^ CL1-5-Luc/GFP-shLacZ or shBMP2 cells) were resuspended in a solution containing 10 µL PBS and 10 µL Basement Membrane Matrix (BD, USA) and directly injected into the right lung via BD Insulin Syringes (30G 3/10 cc, BD, USA). The body weights of xenograft model mice were measured three times per week until sacrifice. All procedures complied with the animal care standards set forth by the guidelines of the NCKU Laboratory Animal Center (Tainan, Taiwan). The whole mouse lung tissue with shLacZ and shBMP2 groups was scanned by TissueFAXS. The total area of metastasis colonies in left lung was quantified using the Image J software(1.53 K).

### IVIS quantification

The metastasis of cancer cells in vivo was assessed using an IVIS (Caliper Life Sciences, USA). Mice were anesthetized by isoflurane and intraperitoneally injected with 100 μl of D-luciferin (PerkinElmer, USA). After 10 min, mice were examined using an IVIS, and the data were analyzed using a Xenogen IVISR Spectrum Noninvasive Quantitative Molecular Imaging System.

### Statistical analysis

Experimental data are presented as the mean ± standard deviation (SD). Differences between groups were compared using the Student's t-test. A *p*-value below 0.05 was considered significant.

## Results

### Higher BMP2 expression levels are observed in lung adenocarcinoma patients with lymph node metastasis than in those without lymph node metastasis

**Figure 1:**
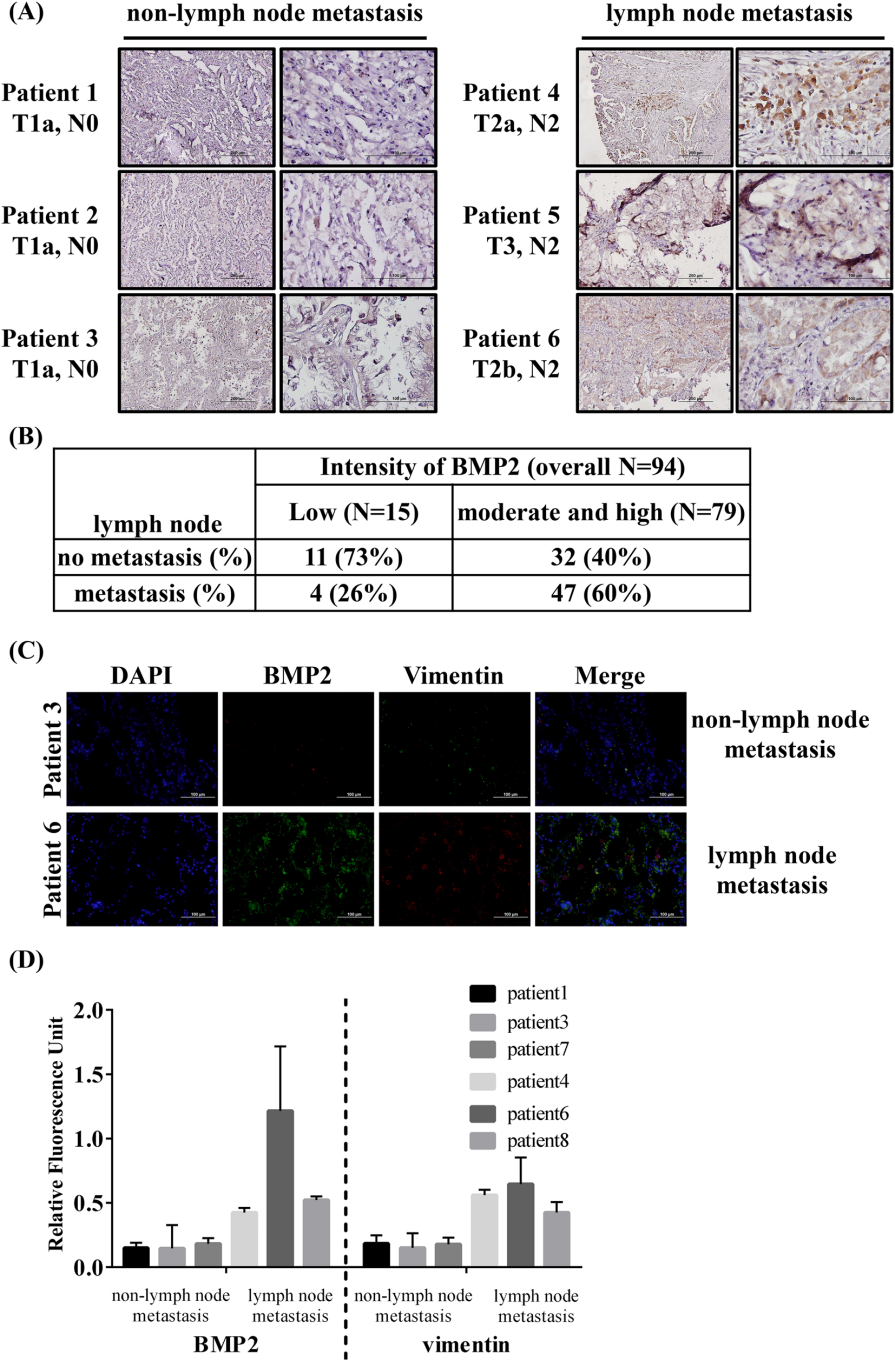
BMP2 is highly expressed in lung adenocarcinoma patients with lymph node metastasis. (**A**) The representative BMP2 immunohistochemistry (IHC) images in tissue samples from patients with and without lymph node metastasis. Slides were observed under 4 × and 40 × magnification. (**B**) BMP2 IHC was analyzed in samples derived from 94 patients with lung cancer. Significance was determined by Fisher’s exact test (*p* = 0.025). (**C**) Representative images of BMP2 and Vimentin immunofluorescence in samples derived from patients with and without lymph node metastasis. Slides were observed under 10 × magnification. (**D**) Quantitative analysis of fluorescence intensity of BMP2 and Vimentin. Analysis of fluorescence intensity was done at the original magnification using ImageJ software. At least three fields were quantified.

Previous studies have reported that patients with NSCLC have higher serum BMP2 levels than healthy individuals^16,18,32,33^. To test whether metastatic cancers express higher BMP2 levels than non-metastatic cancers, we collected lung cancer tissue from National Cheng Kung University Hospital (NCKUH, Taiwan) and separated the samples into two groups. One group contained samples from lung cancer patient with lymph node metastasis, and the other contained samples from lung cancer patient without lymph node metastasis. BMP2 expression levels were assessed as low (N = 15), moderate, or high (N = 79), based on an intensity scale (Fig. 1A,B). There are approximately 60% samples with lymph node metastasis and 40% samples without lymph node metastasis in moderate and high BMP2 expression group (Fig. 1B). In contrast, approximately 26% samples with lymph node metastasis and 73% samples without lymph node metastasis in low BMP2 expression group (Fig. 1B). Our results revealed that samples derived from patients with lymph node metastasis had significantly higher BMP2 expression levels than samples derived from patients without lymph node metastasis (*p*-value = 0.0193, Fig. 1B). Additionally, in samples derived from patients with lymph node metastasis, higher BMP2 expression levels correlated with higher expression levels of the mesenchymal marker vimentin (Fig. 1C,D and Supplementary Fig. 1). Analyzing the median survival times for the low and moderate/high BMP2 expression groups, however, revealed no significant differences in survival times between groups (Table 1 and Fig. 1B). We further analyzed BMP2 levels based age, genderm smoking, and TNM stage and it revealed no significant differences in BMP2 levels (Supplementary Table 1). We also analyzed overall survival based on public databases derived from TCGA and GTEx projects databases^31^. It revealed no significant differences in overall survival with high or low BMP expression (Supplementary Fig. 2).

**Table 1:** Mean survival times among patients with and without high BMP2 expression levels.

	Median for survival time			
	Median	SE	95% Confidence Interval	
			Lower bound	Upper bound
**Intensity of BMP2**				
Low	2797	361.277	2088.898	3505.102
Moderate and high	2799	173.386	2459.163	3138.837
Overall	2799	139.693	2525.201	3072.799

Significance was determined by the Log-rank test (*p* = 0.603).

### BMP2 is highly expressed in more invasive lung adenocarcinoma cells

**Figure 2:**
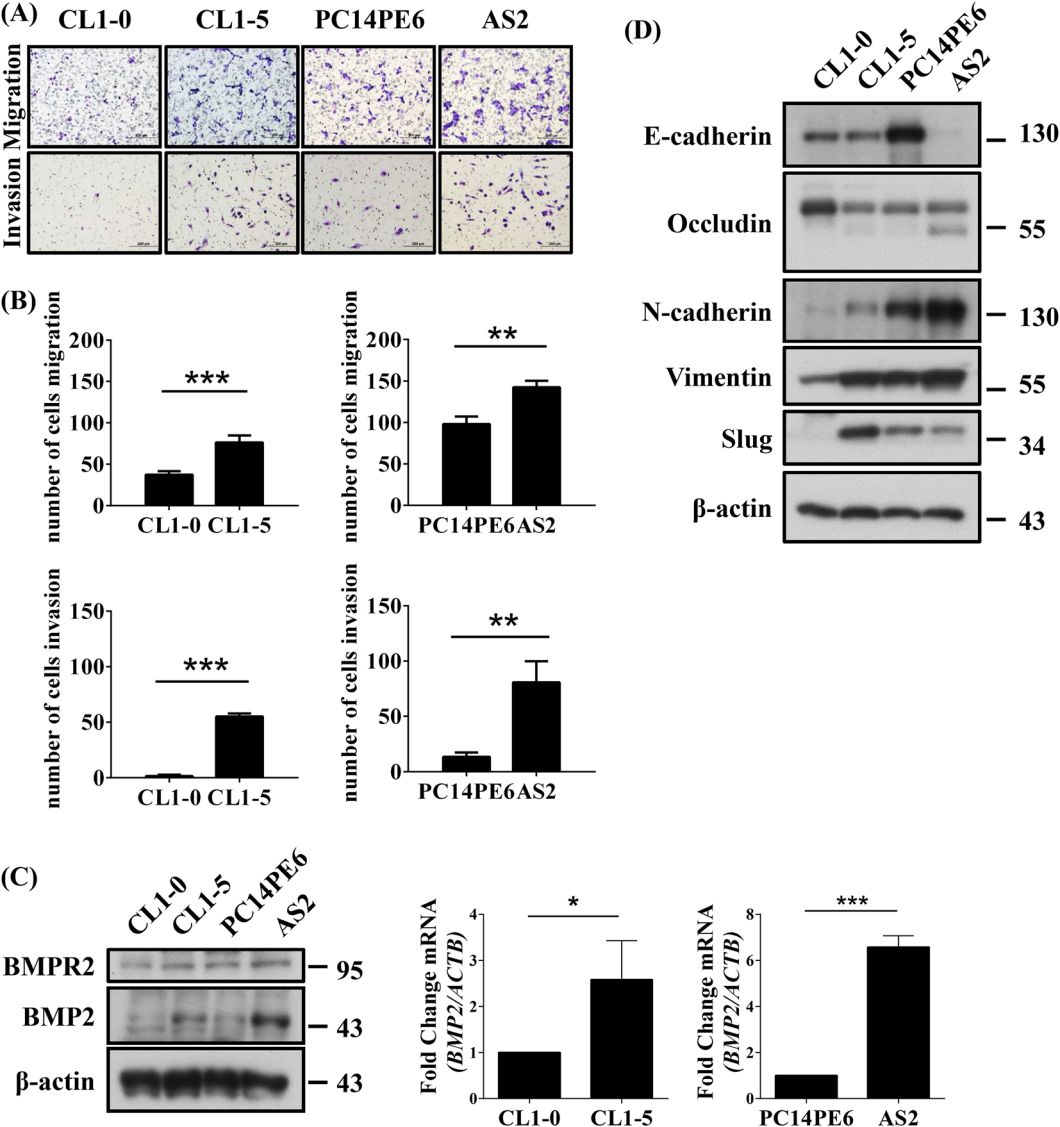
BMP2 is highly expressed in more invasive lung adenocarcinoma cells. (**A**) Migration ability was determined by seeding 5 × 10^4^ cells in a migration chamber without Matrigel coating. Invasion ability was determined by seeding 1 × 10^5^ cells in a migration chamber with Matrigel coating. After 24 h, the cells that successfully migrated through the filter were stained with Liu ‘s solution and counted. Slides were observed under 10 × magnification. (**B**) Quantitative data derived from (**A**). Bar graphs represent the average number of migrated cells ± SD. At least three fields were counted for each condition. (**C**) BMP2 and BMPR2 expression levels were determined by western blotting and qPCR. (**D**) The protein levels of epithelial and mesenchymal marker were analyzed by western blotting. The error bars represent SD, and the *p*-value was determined by Student’s *t* test (*p < 0.05; **p < 0.01; ***p < 0.001).

To test whether BMP2 was associated with the migratory and invasive capabilities of cancer cells, we used the metastatic CL1-5 and AS2 cell lines^28,29^. The highly invasive CL1-5 cell line was derived from the human lung adenocarcinoma CL1-0 cell line by selection through a Transwell invasion chamber^28^. AS2 cells were established from malignant ascites generated in SCID mice following the inoculation of PC14PE6 cells in the peritoneal cavity^29^. AS2 cells are highly invasive, resulting in lung metastases and malignant pleural effusion following intravenous injection. We verified the migratory and invasive abilities of these cell lines using a Transwell assay, without and with Matrigel, respectively. CL1-5 and AS2 cells have enhanced migratory and invasive abilities than CL1-0 and PC14PE6 cells, as previously reported (Fig. 2A,B)^28,29^. Intriguingly, we found that CL1-5 and AS2 have higher BMP2 expression levels than their parental CL1-0 and PC14PE6 cell lines, at both the mRNA and protein levels (Fig. 2C). Additionally, we found that CL1-5 and AS2 cells have higher expression levels of N-cadherin, vimentin, and Slug than the parental CL1-0 and PC14PE6 cells. Conversely, CL1-5 and AS2 have lower expression levels of epithelial marker, such as E-cadherin and occludin than the parental CL1-0 and PC14PE6 cells (Fig. 2D). These data indicate that the more invasive CL1-5 and AS2 cells have higher BMP2 expression levels, which are associated with a mesenchymal phenotype.

### The depletion of BMP2 reduces invasiveness and EMT-associated gene expression levels in lung cancer cells

**Figure 3:**
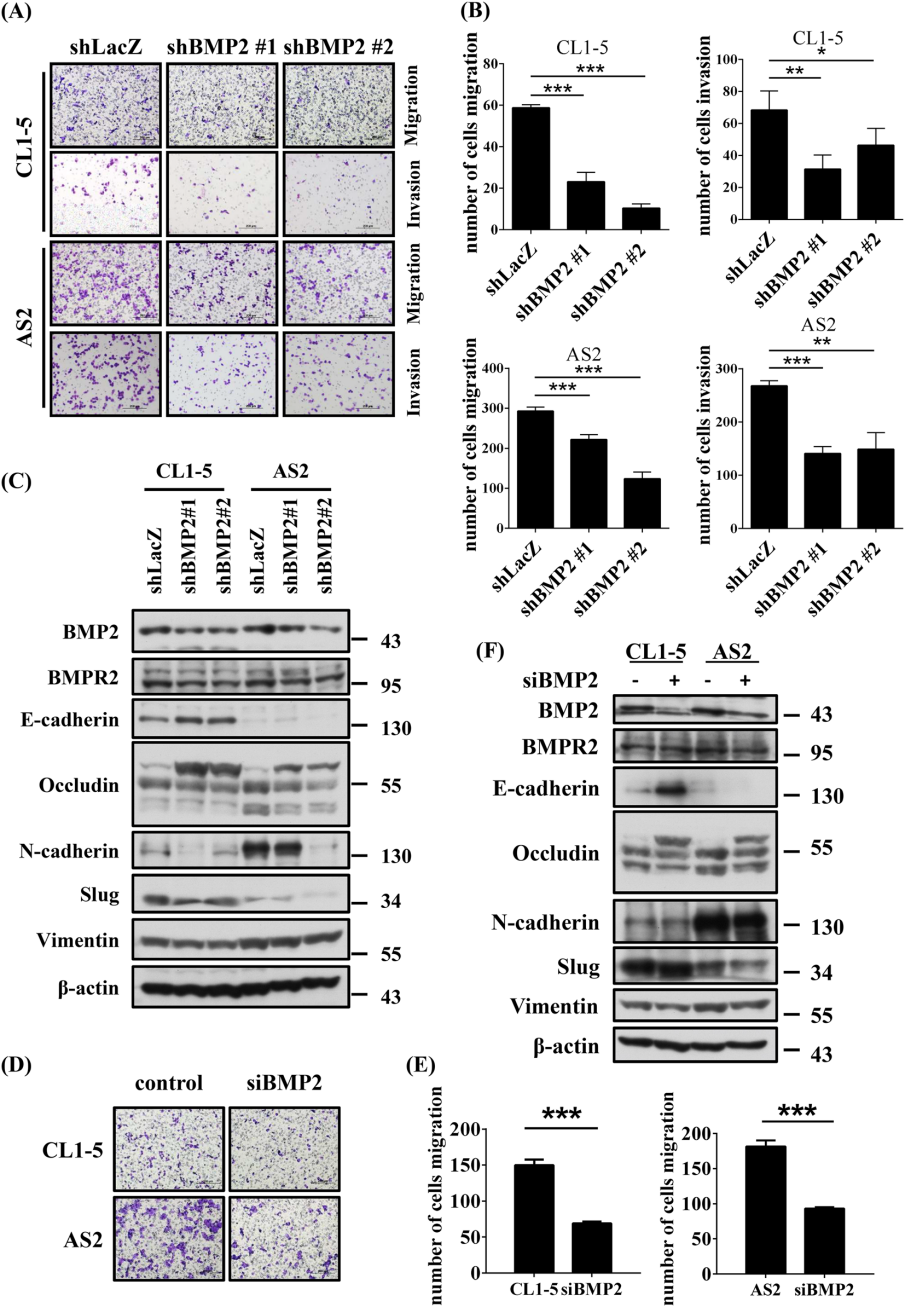
Inhibition of BMP2 reduces invasiveness and downregulates EMT in lung cancer cells. (**A**) The invasion and migration abilities were reduced in BMP2-depleted CL1-5 and AS2 cells. BMP2 expression was depleted by two specific shRNAs. shLacZ is the non-targeting control. Slides were observed under 10 × magnification. (**B**) Quantitative data derived from (**A**). Bar graphs represent the average number of migrated cells ± SD. At least three fields were counted and averaged for each cell line. (**C**) The protein levels of BMP2 and EMT marker were determined by western blotting in CL1-5 and AS2 cells. (**D**) Cell invasion and migration abilities were reduced in BMP2-depleted CL1-5 and AS2 cells. The expression of BMP2 was depleted by siRNA. A non-targeting siRNA was used as a control. Slides were observed under 10 × magnification. (**E**) Quantitative data derived from (**D**). Bar graphs represent the average number of migrated cells ± SD. At least three fields were measured in each cell line. (**F**) The expression levels of BMP2 and EMT markers were determined by western blotting. The *p*-value was determined by Student’s *t* test. (*p < 0.05, **p < 0.01, ***p < 0.001).

To test whether BMP2 promotes lung adenocarcinoma mobility through the epithelial-to-mesenchymal transition (EMT), we depleted the expression of BMP2 using lentiviral-mediated shRNA delivery to CL1-5 and AS2 cells. As shown in Fig. 3A,B, BMP2 depletion reduced the migratory and invasive abilities of CL1-5 and AS2 cell lines. Additionally, we found that BMP2 depletion altered the cell morphology, resulting in more rounded cells compared with shLacZ control cells (Supplementary Fig. 3). Furthermore, BMP2 depletion reduced the expression of mesenchymal markers, such as N-cadherin and Slug, but increased the expression of epithelial markers, such as E-cadherin and occludin (Fig. 3C). We verified these results using siRNA to deplete BMP2 expression. Consistently, the BMP2 depletion using siRNA reduced cell migration and the expression levels of N-cadherin, Slug, and vimentin but increased the expression levels of E-cadherin and occludin (Fig. 3D–F). Our results indicate that BMP2 promotes EMT in lung adenocarcinoma cells.

### BMP2 activates the SMAD1/5/8 pathway and enhances cell migration in lung cancer cells

**Figure 4:**
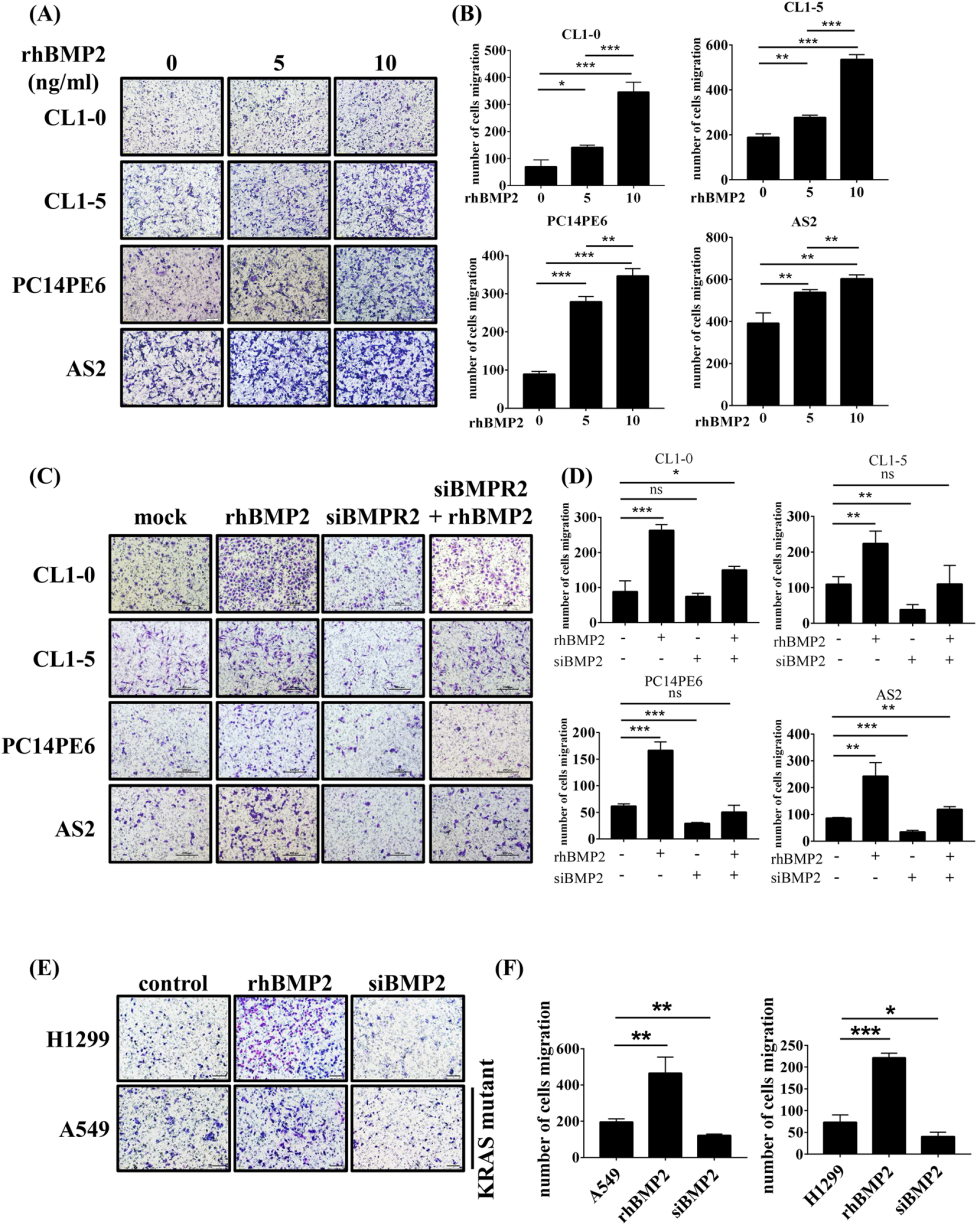
Recombinant BMP2 treatment increases cell migration in human lung cancer cells. (**A**) The cell migration ability was determined by seeding cells in a migration chamber without Matrigel coating. Cells that migrated through the filter were stained with Liu ‘s solution and counted. Slides were observed under 10 × magnification. Cells were treated with 0, 5, 10 ng/ml recombinant human BMP2 (rhBMP2) in culture medium. (**B**) Quantitative data derived from (**A**). Bar graphs represent the average number of migrated cells ± SD. At least three fields were counted for each cell line following rhBMP2 treatment. (**C**) Representative images of cell migration of each cell line treated with 20 ng/ml rhBMP2 with or without siBMPR2. Slides were observed under 10 × magnification. (**D**) Quantitative data derived from (**C**). Bar graphs represent the average number of migrated cells ± SD. At least three fields were counted for each cell line. (**E**) Representative images of cell migration for each cell line. A549 and H1299 cells were treated with 10 ng/ml rhBMP2 or transfected with siBMP2. Slides were observed under 10 × magnification. (**F**) Quantitative data derived from (**E**). Bar graphs represent the average number of migrated cells ± SD. At least three fields were measured for each condition. The *p*-value was determined by Student’s *t* test (*p < 0.05, **p < 0.01, ***p < 0.001).

To further test the role of BMP2 in lung cancer metastasis, we treated CL1-0, CL1-5, PC14PE6, and AS2 cells with rhBMP2 to activate the BMP signaling pathway. All cell lines treated with rhBMP2 showed a dose-dependent increase in migratory ability (Fig. 4A,B). Previous studies have shown that BMP2 binds to BMPR2 to activate the SMAD1/5/8 signaling pathway in smooth muscle cells^34^. To test whether the observed enhancement in migratory ability was mediated through the BMPR2 receptor, we depleted BMPR2 expression using siRNA. The depletion of BMPR2 reduced cell migration, even in the presence of rhBMP2, in all cell lines, suggesting that enhanced cell migration is mediated by BMP2-BMPR2 signaling (Fig. 4C,D). Treatment with rhBMP2 also induced SMAD1/5 phosphorylation, which increased in a dose-dependent manner in all cell lines (Supplementary Fig. 4A). Conversely, the depletion of BMPR2 reduced SMAD1/5 phosphorylation levels (Supplementary Fig. 4B), indicating that SMAD1/5 phosphorylation may be involved in cell migration and invasiveness. CL1-0, CL1-5, PC14PE6, and AS2 are all *KRAS*-wild type cells. To test whether *KRAS* plays a role in the BMP signaling pathway, particularly BMP2-mediated cell migration and invasiveness, we examined the effects of rhBMP2 treatment in the *KRAS*-mutant cell lines A549 and H1299. In the *KRAS*-mutant cells, rhBMP2 treatment induced cell migration (Fig. 4E,F), whereas siRNA-mediated BMP2 depletion reduced cell migration, as measured using the Transwell assay (Fig. 4E,F). These results suggest that BMP2-mediated cell migration occurs independently of the *KRAS* signaling pathway. Similar to the results observed in CL1-0, CL1-5, PC14PE6, and AS2 cells, rhBMP treatment induced SMAD1/5 phosphorylation in A549 and H1299 cells (Supplementary Fig. 4C). These results demonstrate that the BMP signaling pathway activates SMAD1/5 phosphorylation and enhances cell migration independent of the *KRAS* signal pathway.

### The inhibition of SMAD1/5/8 signaling attenuates cell migration in lung cancer cells

**Figure 5:**
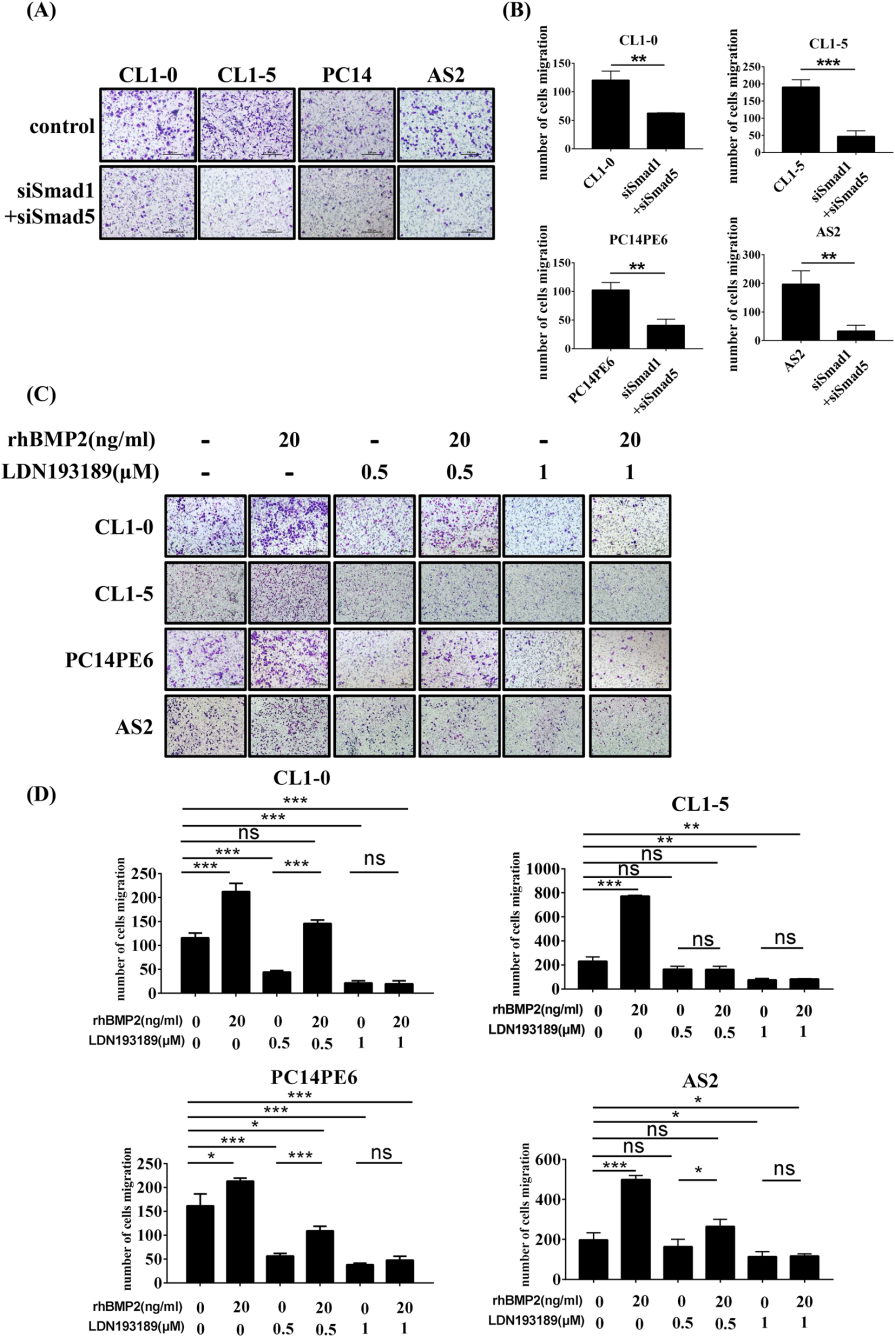
The depletion or inhibition of SMAD pathway attenuates lung cancer cell migration. (**A**) The cell migration abilities were reduced in SMAD1/5-depleted cells. Slides were observed under 10 × magnification. (**B**) Quantitative data derived from (**B**). Bar graphs represent the average number of migrated cells ± SD. At least three fields were counted for each condition. (**C**) Representative images of cell migration for each cell line. Cells were treated with 20 ng/ml rhBMP2 with or without BMP signaling inhibitor LDN193189 as indicated. Slides were observed under 10 × magnification. (**D**) Quantitative data derived from (**C**). Bar graphs represent the average number of migrated cell ± SD. At least three fields were counted for each condition. The *p*-value was determined by Student’s *t* test (*p < 0.05, **p < 0.01, ***p < 0.001).

To test whether SMAD1/5/8 has profound effects on cell migration, we used siRNA to deplete SMAD1/5 expression (Supplementary Fig. 5A). As shown in Fig. 5A,B, the depletion of SMAD1/5 significantly reduced cell migration in CL1-0, CL1-5, PC14PE6, and AS2 cells. Treating cells with a BMP/SMAD inhibitor LDN193189 to block the activation of BMP/SMAD signaling pathway resulted in significantly reduced SMAD1/5 phosphorylation levels (Supplementary Fig. 5B) and cell migratory ability, even in the presence of rhBMP2 (Fig. 5C,D). The results suggest that BMP2-BMPR2 binding induces cell migration and invasiveness through the activation of the SMAD1/5 pathway in lung cancer cells.

### BMP2 depletion decreases metastasis in an orthotopic mouse model

**Figure 6:**
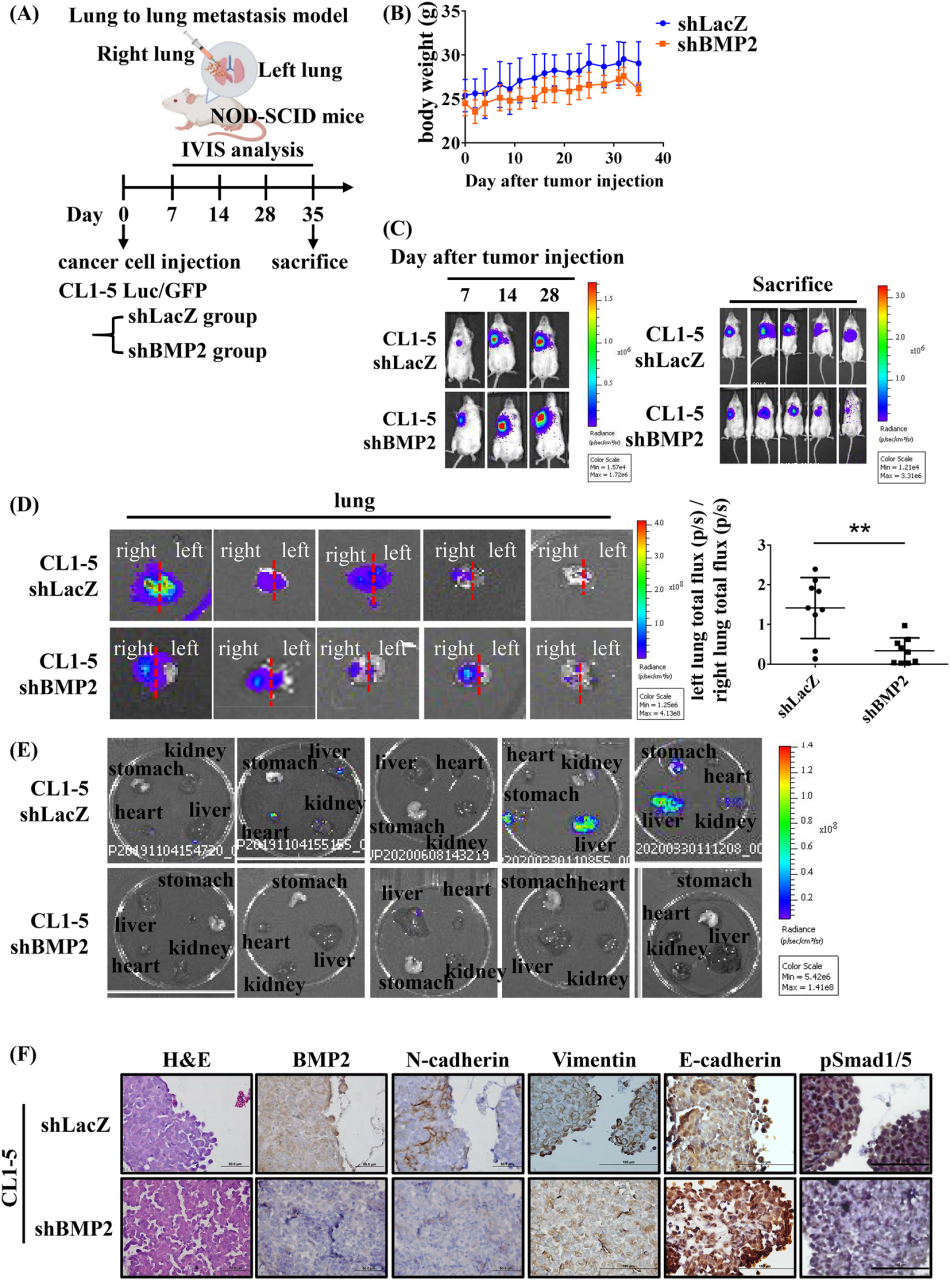
Inhibition of BMP2 decreased metastasis in a CL1-5 orthotopic mouse model. (**A**) A schematic diagram showing the animal experiment. CL1-5 cells stably expressing shLacZ or shBMP2 Luc/GFP were injected into the right lungs of NOD-SCID mice. IVIS analysis was conducted at 7, 14, 28, and 35 days after injection. (**B**) The body weights of mice were measured three times per week after tumor injection. (**C**) Metastasis in shLacZ and shBMP2-CL1-5-Luc/GFP groups was measured by IVIS until sacrifice. (**D**,**E**) Metastastic lesions in the lungs (**D**) and nearby organs (**E**) were analyzed by IVIS after sacrifice. (**F**) Hematoxylin and eosin (H&E) and IHC stains in mouse right lung tissue for the indicated proteins. Slides were observed under 40 × magnification. Error bars represent SD, and *p*-value was determined by Student’s *t* test (*p < 0.05, **p < 0.01, ***p < 0.001).

To examine whether BMP2 depletion reduces cancer metastasis in vivo, we used 7–8 week old NOD-SCID mice to generate a lung-to-lung orthotopic metastasis mouse model. We generated shLacZ (control) and shBMP2 (BMP2 knockdown) CL1-5 cells labeled with luciferase (Luc) and GFP and engrafted 2 × 10^5^ cells onto the right lungs of NOD-SCID mice by orthotopic injection (Fig. 6A). Tumor growth was monitored using an in vivo imaging system (IVIS). No significant differences in body weight were observed between shLacZ and shBMP2 xenografted model mice (Fig. 6B). Significantly, the shBMP2 group demonstrated reduced lung-to-lung metastasis compared with the shLacZ group, as measured by IVIS (Fig. 6C). As shown in Fig. 6D, the shBMP2 group had lower numbers of metastatic cancer cells in the left lung than the shLacZ group. We also evaluated nearby organs such as the heart, liver, kidney, and stomach, and discovered that BMP2-depleted cancer cells reduced metastasis to nearby organs (Fig. 6E). Previous studies have demonstrated that BMP2 induced inflammation with dose dependent of BMP2 treatment^35,36^. Consistent with previous findings, we found that the left lung tissue showed more macrophages in the shLacZ group compared to shBMP2 groups, as assessed by H&E staining (Supplementary Fig. 6), indicating more severe inflammation in shLacZ groups than the shBMP2 groups. In addition, we found that tumor cells expressed higher levels of BMP2, N-cadherin, Vimentin and p-SMAD1/5 in the shLacZ group than in the shBMP2 group by IHC both in primary tumors (right lung) and metastatic tumors (left lung) (Fig. 6F and supplementary Fig. 7A,B). The area of metastatic colonies in the left lung were larger in shLacZ than in shBMP2 (supplementary Fig. 8). Our data clearly demonstrated that the depletion of BMP2 significantly reduces metastasis in vivo.

## Discussion

In this study, we provide in vivo evidence suggesting that BMP2 promotes NSCLC cell metastasis in an orthotopic mouse model. The overexpression of BMP2 was also observed in clinical samples. Using the highly metastatic human CL1-5 and AS2 cell lines as in vitro models, we also found that highly metastatic cells express higher BMP2 levels than their parental cell lines, CL1-0 and PC14PE6, respectively. The depletion of either BMP2 or its receptor, BMPR2, significantly reduced cell migratory and invasive abilities. We further demonstrated that BMP2 induces SMAD1/5/8 phosphorylation, independent of the *KRAS* signaling pathway. In addition, the depletion of SMAD1/5/8 or inhibition of SMAD1/5/8 by LDN193189 significantly reduced cell migration. Therefore, targeting the BMP2 signaling pathway may represent a potential therapeutic strategy for treating patients with metastatic NSCLC.

Previous studies have shown that BMP2 signaling enhances bone metastasis of mice Lewis lung carcinoma and breast cancer cells using xenografted mouse model. Lung metastasis of breast cancer cells by BMP2 signaling pathway is also reported in the mouse model. Different from previous studies, we used the metastatic human lung adenocarcinoma cell lines CL1-5 which were generated through selection using a transwell invasion chamber. We observed that CL1-5 had higher expression levels of BMP2 and EMT markers (N-cadherin, Vimentin, and Slug). BMP2 depletion reduced migration and invasion ability and simultaneously the expression levels of N-cadherin, vimentin, and Slug. These results suggest that BMP2 promotes cell migration and invasion. Consistently, our lung to lung metastasis mouse model revealed that CL1-5 shLacZ control cells are highly mestastatic, invading to nearby heart, liver, kidney, and stomach. BMP2 depletion reduced metastastic ability of CL1-5. However, we did not isolate bone and lymph node metastasis in our mouse model. Further studies will be conducted to test it. In addition, clinical specimens derived from our university hospital (NCKUH) also revealed that BMP2 expression levels correlated with lymph node metastasis. However, BMP2 expression levels revealed no significant differences in survival. Further analyzing these databases based on age, gender, smoking, and TNM stage also revealed no significant impact of BMP2. Consistent with our results, public databases derived from TCGA and GTEx projects also revealed no significant impact of BMP2 in survival. To explain why BMP2 expression levels in cancer cells did not correlate with prognosis, we have to consider tumor microenvironment, which are composed of cancer-associated fibroblasts that support tumor epithelial growth, invasion, and therapeutic resistance. Cooperative interaction among these fibroblasts and tumor cells contribute to cancer progression. Previous studies have revealed that lung tumor-associated osteoblast, mouse embryonic fibrobast (MEF), or α-Sma + marked fibroblast cells secret much more BMP2 than carcinoma cells per se, indicating that stroma fibroblast cells might be major source of BMP2, promoting tumor cell migration and invasion^20,24^. Therefore, our clinical study and *in-silico* analysis based on cancer cells only could be insufficient, because we omit the effects from surrounding cancer-associated fibroblasts. Indeed, it has been shown that high BMP2 derived from stroma cells correlated with poor outcome in lung carcinoma^37^. Consistent with this idea, several studies have revealed that serum BMP2 levels are upregulated in patients with advanced NSCLC and corelated with poor survival^15,38^. Therefore, we have to consider BMP2 levels in stroma fibroblasts that could use as a prognosis marker.

Whether BMP signaling promotes metastasis or serves as a barrier to metastasis remains controversial^4^. Several lines of evidence have revealed that BMP signaling functions as a barrier against tumorigenesis and metastasis. Mutations in BMP receptors and SMAD are associated with JP, which has been linked to an enhanced risk of developing cancer. In prostate cancer cell lines and PTEN-null mouse model of prostate cancer, SMAD4 loss has been shown to promote invasion and metastasis^39^. The downregulation of SMAD4 has been reported in patients with metastatic prostate cancer^39^. Consistently, the BMP antagonists COCO and NOG, which inhibit BMP signaling, are able to promote breast cancer metastasis^10,11^. However, other studies, including our study, demonstrate that the activation of BMP signaling promotes lung cancer cell metastasis^13,14,19–21^. The outcome of BMP signaling pathway activation likely depends on the combination of BMP molecules and receptors involved, in addition to genetic background. The timing of BMP signaling is also important. Taken together, all current understanding of BMP signaling suggests that BMP signaling may function as a barrier, preventing tumorigenesis in normal tissues. However, once the barrier has been breached and tumorigenesis is initiated, BMP signaling supports cancer progression. The levels of BMPs also correlate with more advanced tumor grades, further supporting this theory^40^.

Because high BMP2 levels are associated with more advanced and highly metastatic NSCLC, targeting the BMP2 signaling pathway may serve as a therapeutic strategy for treating patients with metastatic NSCLC.

## Supplementary Information

Supplementary Information 1.

Supplementary Information 2.

Supplementary Information 3.

## Data Availability

Materials and cell lines are available upon request. The data associated with this study are presented in the paper. All data are available from the corresponding author upon reasonable request.
