# Supplementary material for: BMP2 promotes lung adenocarcinoma metastasis through BMP receptor 2-mediated SMAD1/5 activation
Source: Sci Rep. 2022 Sep 29;12:16310. doi: 10.1038/s41598-022-20788-2 (PMC9522928; doi:10.1038/s41598-022-20788-2)
Supplement: Supplementary file 1 — Supplementary Information 1. [file 41598_2022_20788_MOESM1_ESM.pdf]

Supplementary Figure 1.

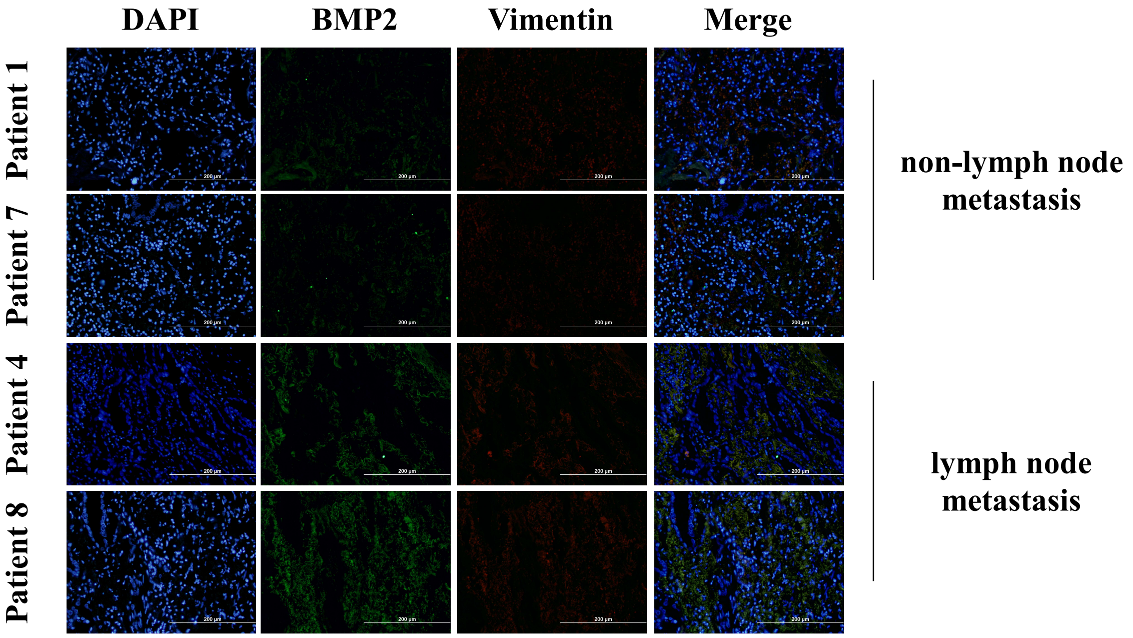

Supplementary Figure 1. Representative images of BMP2 and Vimentin immunofluorescence staining.

Samples derived from four patients with and without lymph node metastasis were stained with specific antibodies as indicated. Slides were observed under 10x magnification.

Supplementary Figure 2.

(A)

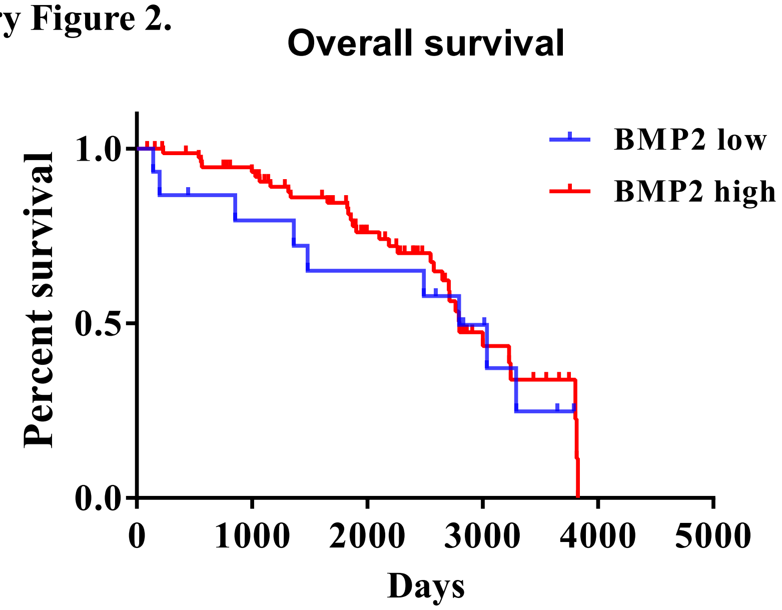

(B)

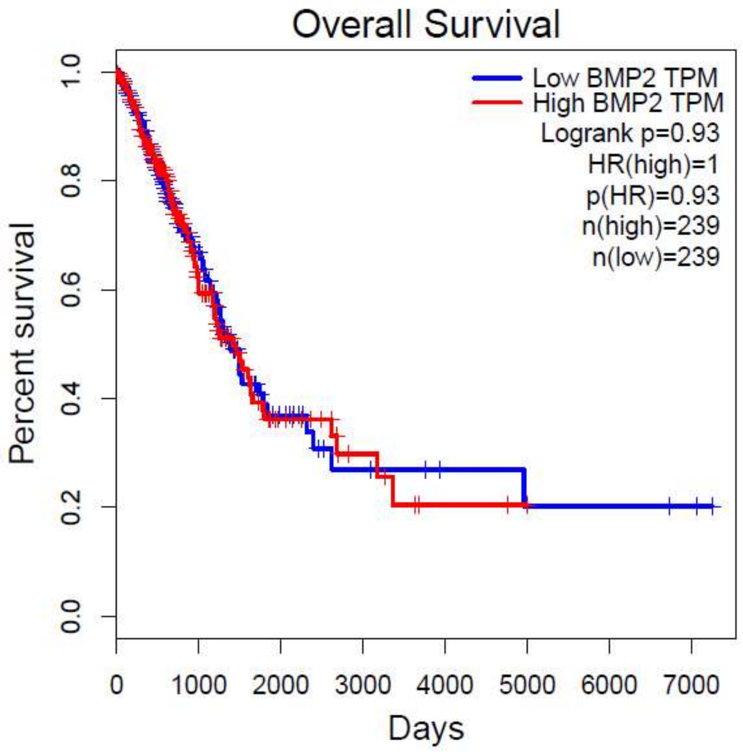

Supplementary Figure 2. Kaplan-Meier curves of overall survival in patients with high BMP expression (red) and low BMP expression (blue)

(A) Overall survival of 94 patients derived from National Cheng Kung University Hospital (Tainan, Taiwan), Log-rank test (p= 0.603) (B) Overall survival derived from public database with lung adenocarcinoma group (http://gepia.cancer-pku.cn/index.html). HR: hazard ratio. n (high): patient numbers of high BMP2 expression. n (low): patient number of low BMP2 expression.

Supplementary Figure 3.

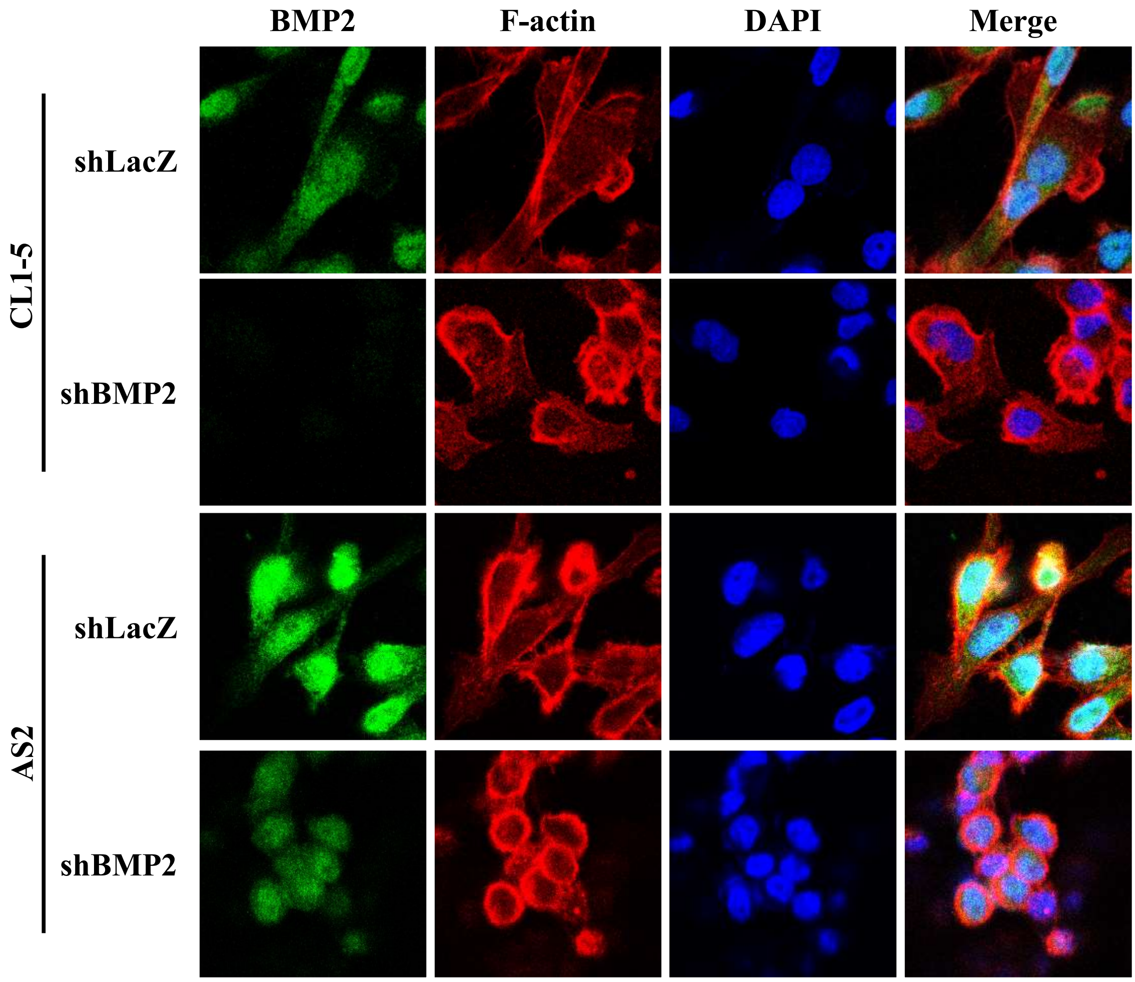

Supplementary Figure 3. Depletion of BMP2 shifts cell morphology toward epithelial cell types. The cell morphology of BMP2-depleted CL1-5 and AS2 cells. Images were observed under 20x magnification. Immunostaining of each protein was observed under confocal microscopy.

Supplementary Figure 4.

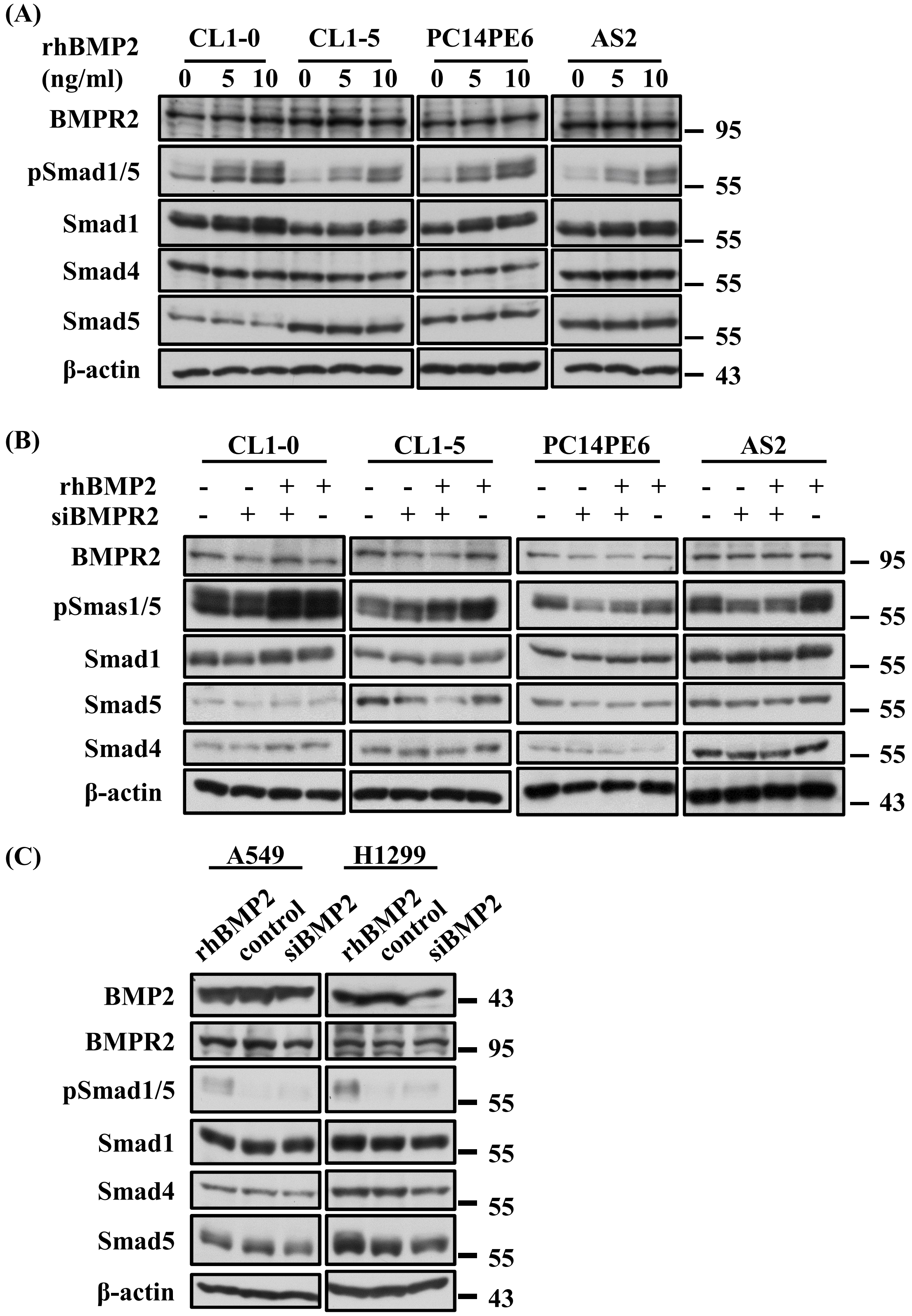

Supplementary Figure 4. Recombinant BMP2 treatment increases p-Smad1/5 expression.
(A) Protein levels were determined by western blotting. Cells were treated with 0, 5, and 10 ng/ml rhBMP2. (B) Protein levels were determined by western blotting for cells treated with rhBMP2 with or without siBMP2, as indicated. (C) Protein levels were determined by western blotting. Cells were treated with rhBMP2 or siBMP2.

Supplementary Figure 5.

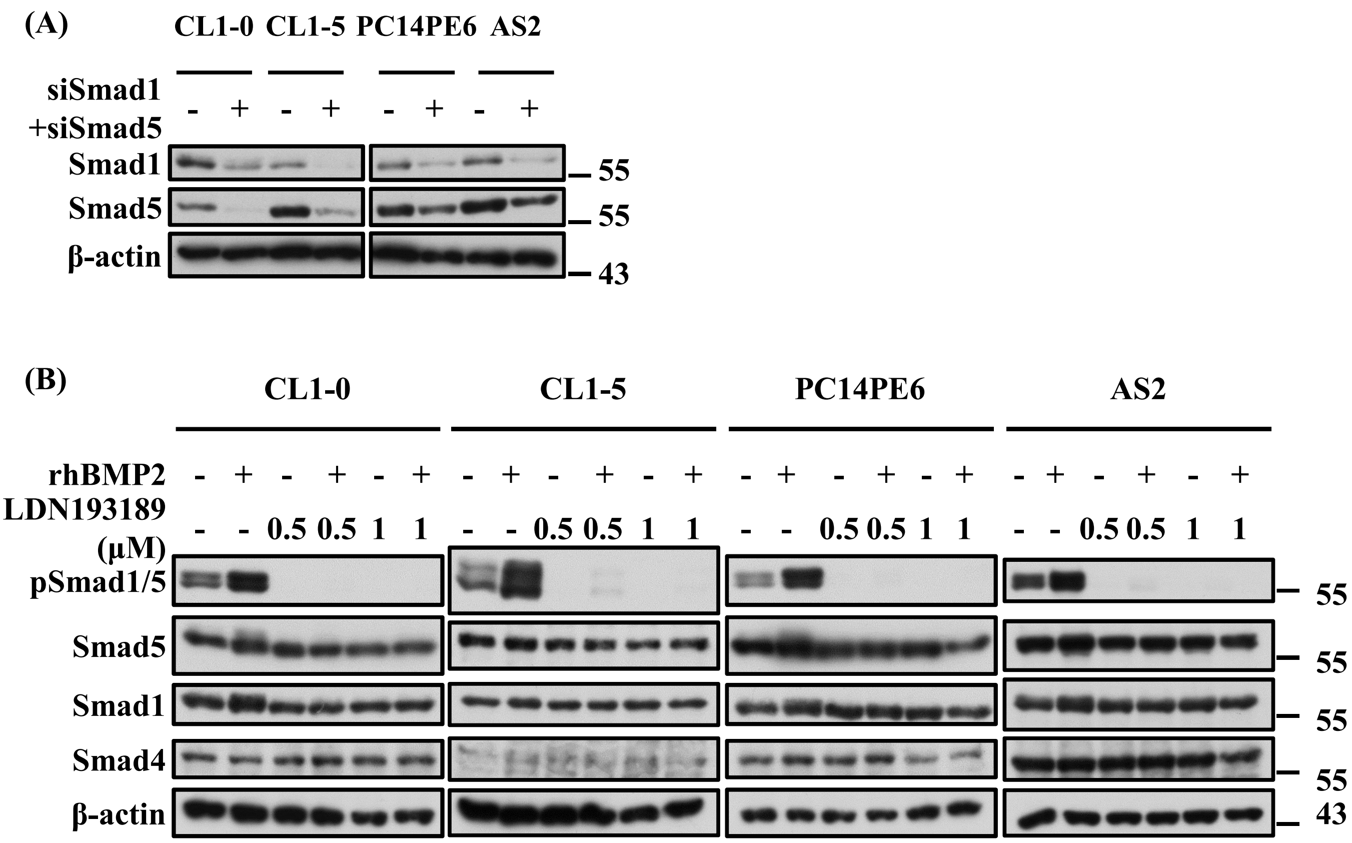

Supplementary Figure 5. The western blotting analysis with the depletion or inhibition of SMAD pathway treatment.

(A) Protein levels of SMAD1 and SMAD5 were determined by western blotting. The expression of SMAD1 and SMAD5 was depleted by specific siRNAs. (B) Protein levels were determined by western blotting. Cells were treated with 20 ng/ml rhBMP2 with or without BMP signaling inhibitor LDN193189 as indicated.

Supplementary Figure 6.

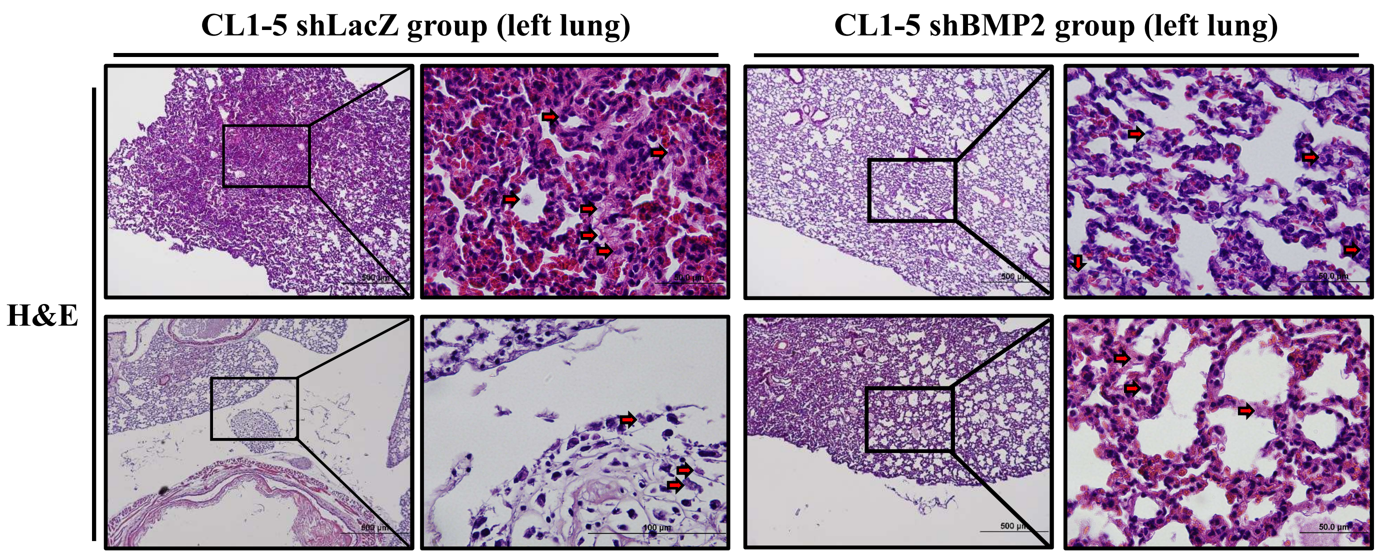

Supplementary Figure 6. H&E stains in mouse left lung tissue.
The CL1-5 cells stably expressing shLacZ and shBMP2 were orthotopically injected into the right lungs of SCID mice. H&E staining was performed in mouse left lung tissues after sacrifice. Macrophage is indicated by red arrow

Supplementary Figure 7.

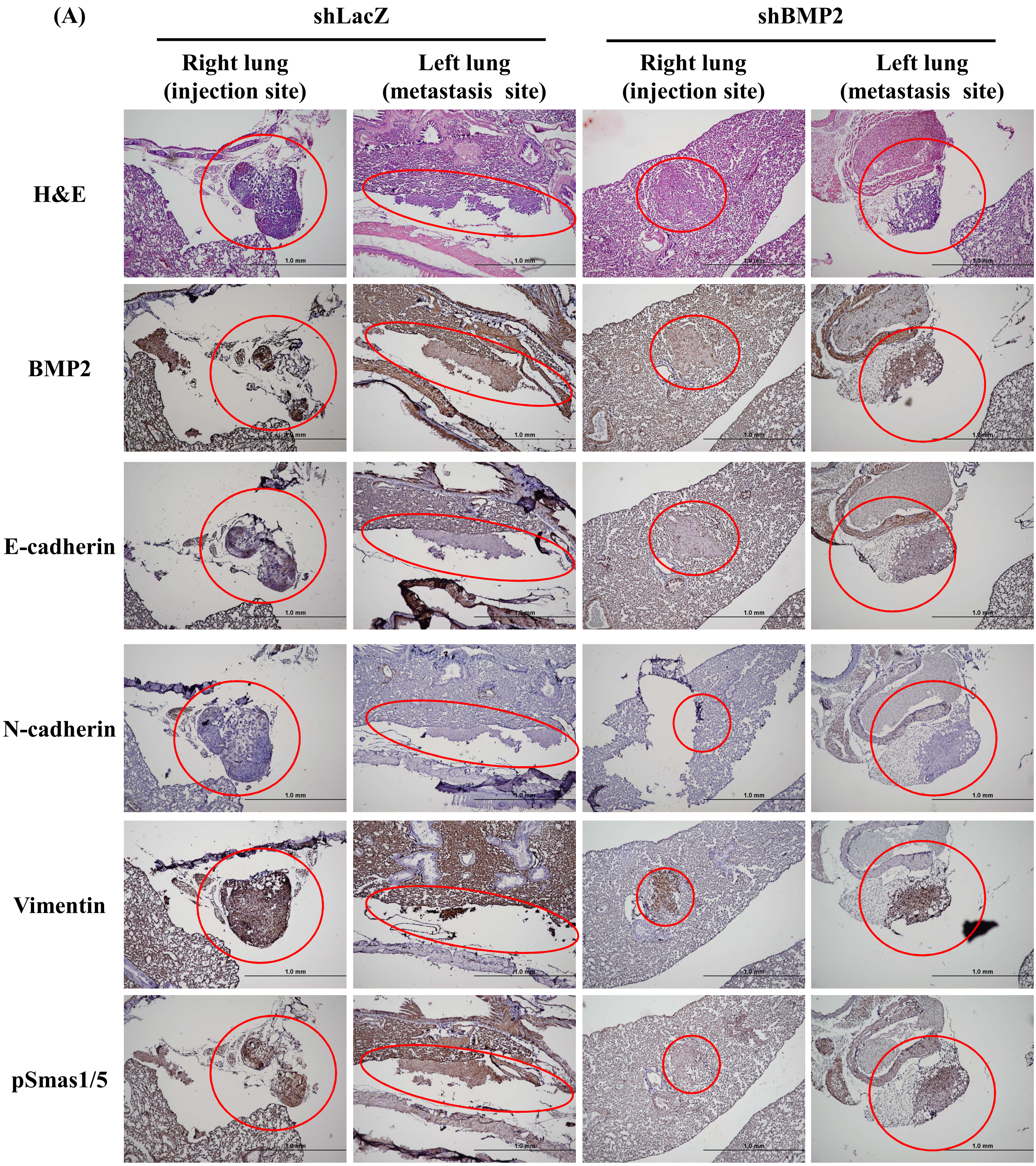

Supplementary Figure 7.

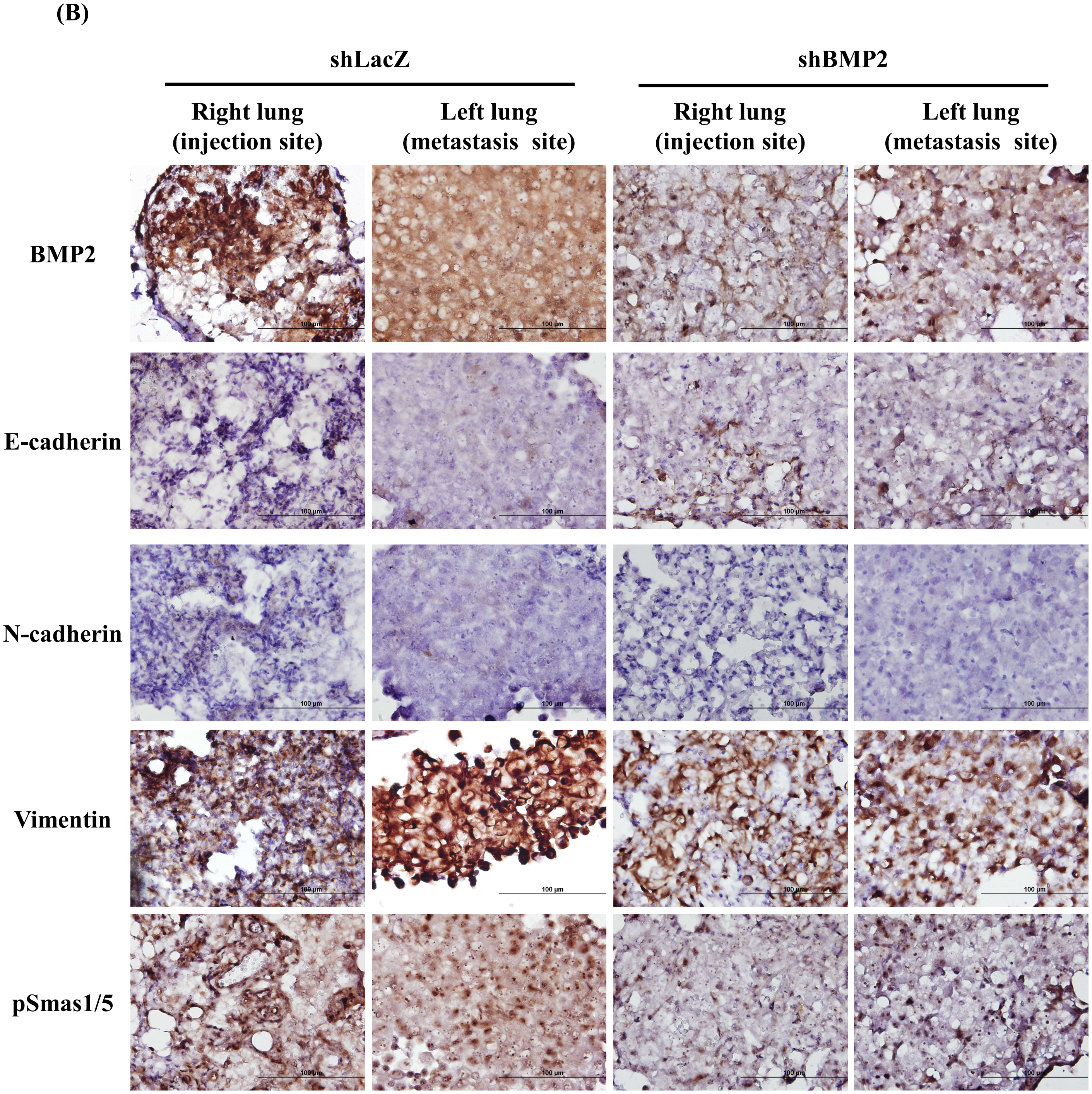

Supplementary Figure 7. Hematoxylin and eosin (H&E) and IHC staining in mouse right and left lung tissues.

The CL1-5 cells stably expressing shLacZ and shBMP2 were orthotopically injected into the right lungs of SCID mice. The tumor is labelled by red cycle. Representative images of BMP2, E-cadherin, N-cadherin, pSmas1/5, and Vimentin IHC staining in samples derived from supplementary figure 8. (A) Slides were observed under 4x magnification. (B) Slides were observed under 40x magnification.

Supplementary Figure 8.

(A)

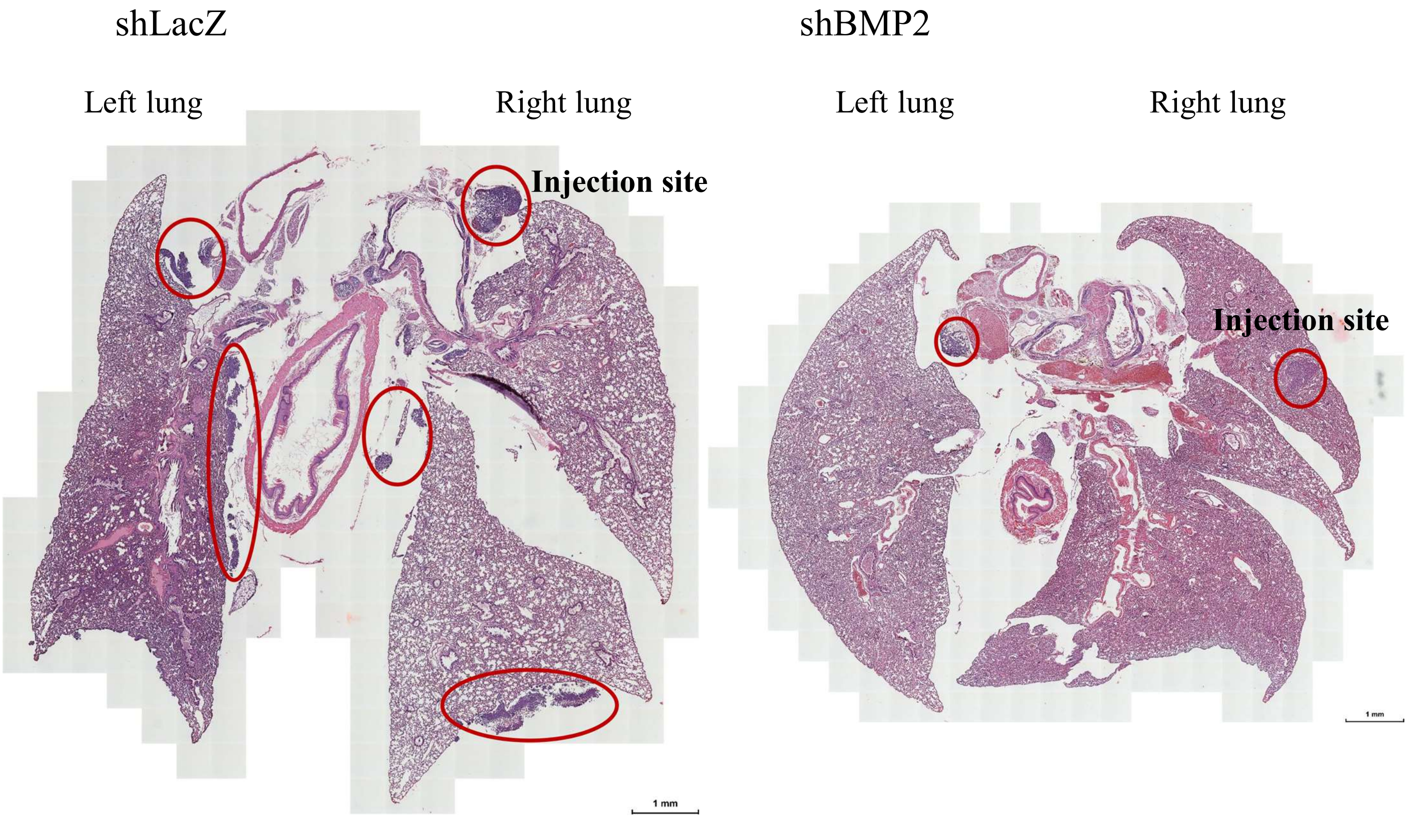

(B)

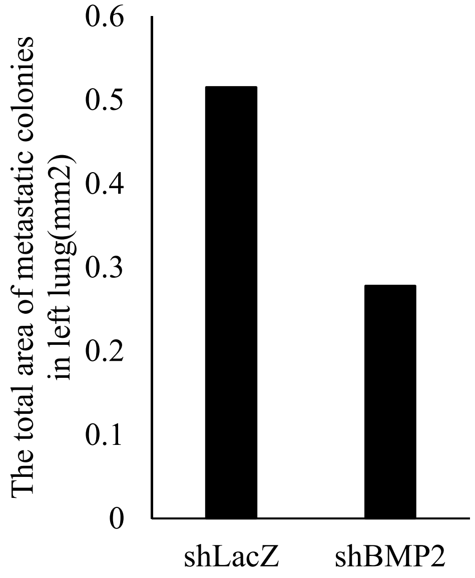

Supplementary Figure 8. Hematoxylin and eosin (H&E) in mouse whole lung tissue.
(A) The whole mouse lung tissue with shLacZ and shBMP2 groups was scanned by TissueFAXS. Tumor metastatic colonies were marked by red cycles. (B) The total area of metastasis colonies derived from (A) in left lung was quantified using the Image J software.
