# Supplementary material for: BMP2 promotes lung adenocarcinoma metastasis through BMP receptor 2-mediated SMAD1/5 activation
Source: Sci Rep. 2022 Sep 29;12:16310. doi: 10.1038/s41598-022-20788-2 (PMC9522928; doi:10.1038/s41598-022-20788-2)
Supplement: Supplementary file 3 — Supplementary Information 3. [file 41598_2022_20788_MOESM3_ESM.pdf]

Western blots-original data files. Full Blots for all Figures. Some blots were cut before hybridization with antibodies. The protein markers were from Thermo Scientific PageRuler Prestained Protein Ladder (Thermo) #26616LCS. These images were acquired by the X-ray film.

Fig 2.C

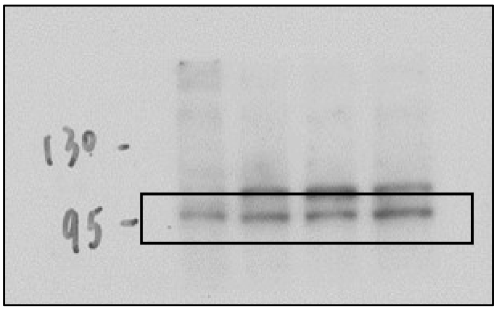

Fig 2C BMPR2

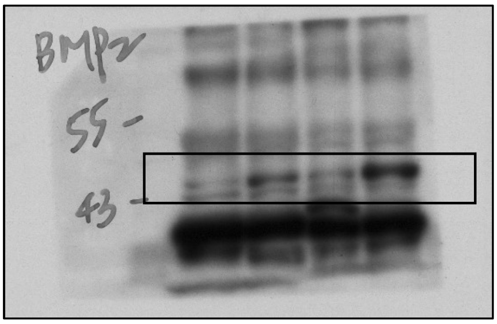

Fig 2C BMP2

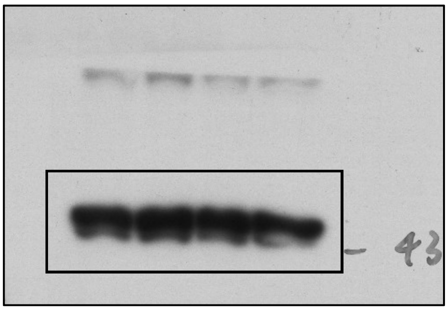

Fig 2C β-actin

Fig 2D

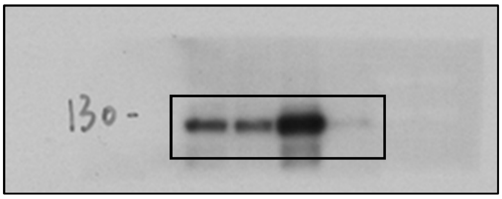

Fig 2D E-cadherin

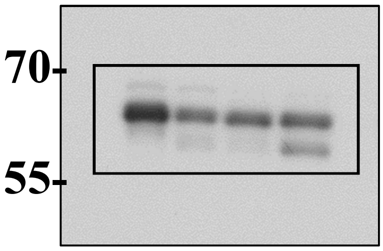

Fig 2D Occludin

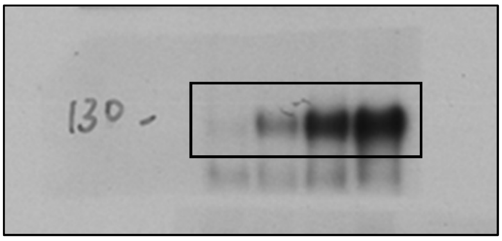

Fig 2D N-cadherin

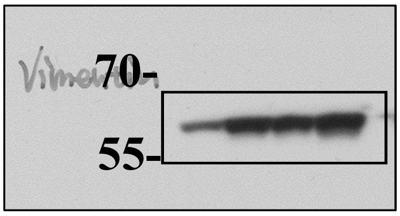

Fig 2D Vimentin

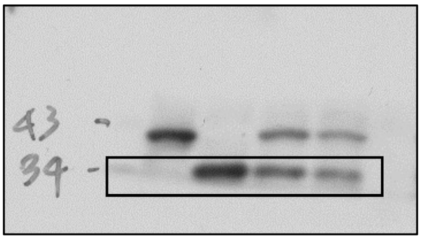

Fig 2D Slug

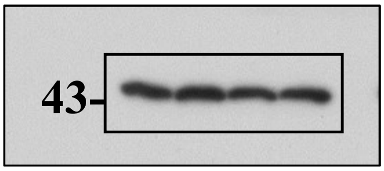

Fig 2D β-actin

Fig 3C

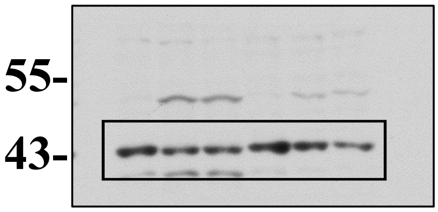

Fig 3C BMP2

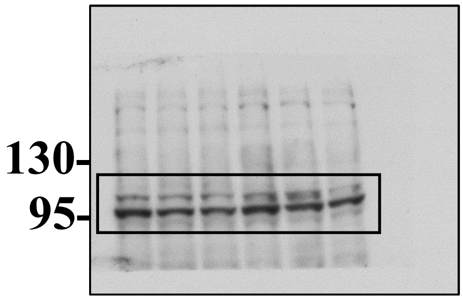

Fig 3C BMPR2

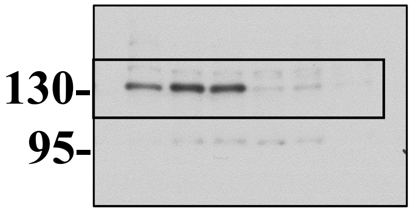

Fig 3C E-cadherin

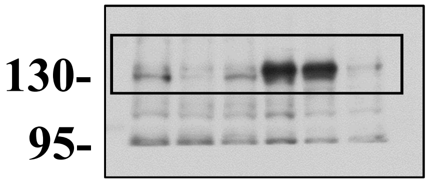

Fig 3C N-cadherin

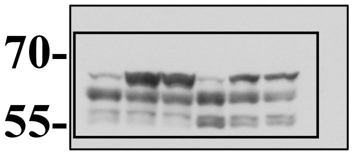

Fig 3C Occludin

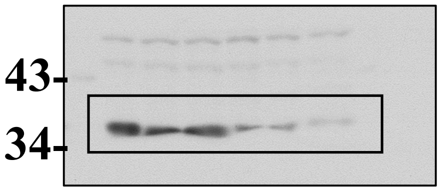

Fig 3.C Slug

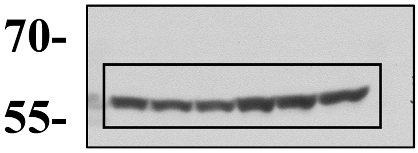

Fig 3C vimentin

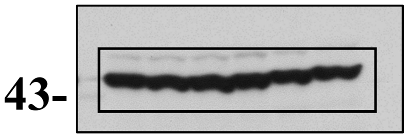

Fig 3C β-actin

Fig 3F

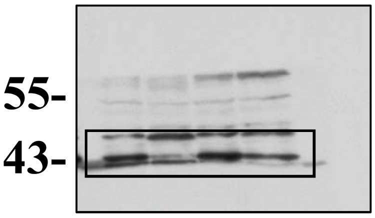

Fig 3F BMP2

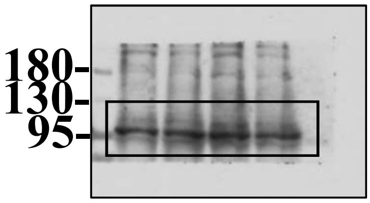

Fig 3F BMPR2

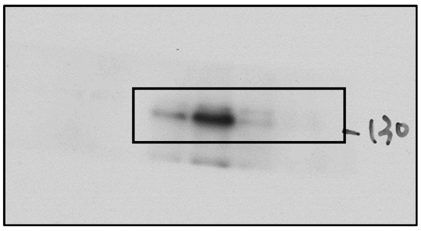

Fig 3F E-cadherin

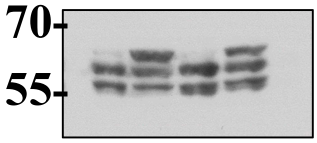

Fig 3F Occludin

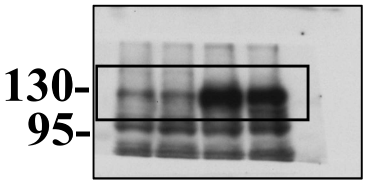

Fig 3F N-cadherin

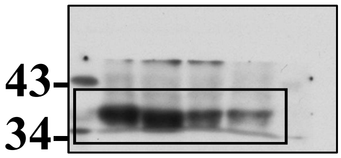

Fig 3F Slug

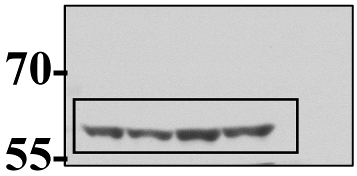

Fig 3F Vimentin

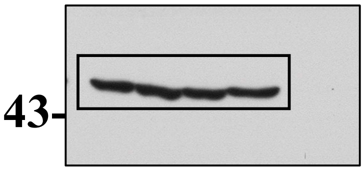

Fig 3F β-actin

Supplementary Figure 4A

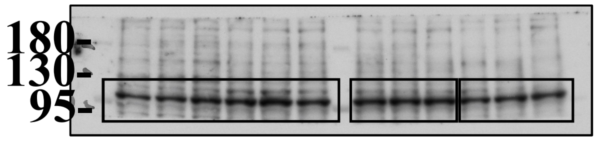

Supplementary Fig 4A BMPR2

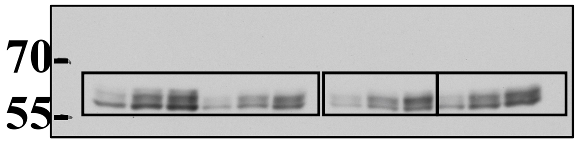

Supplementary Fig 4A pSmad1/5

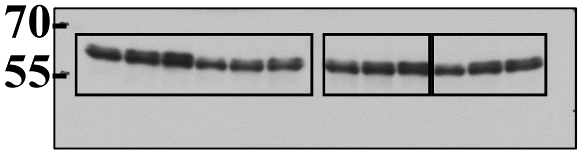

Supplementary Fig 4A Smad1

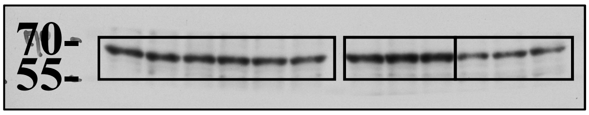

Supplementary Fig 4A Smad4

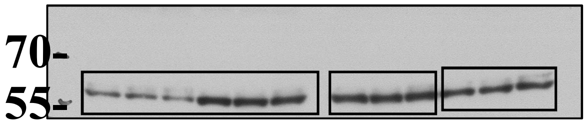

Supplementary Fig 4A Smad5

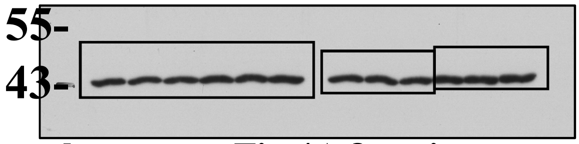

Supplementary Fig 4A β-actin

Supplementary Figure 4B

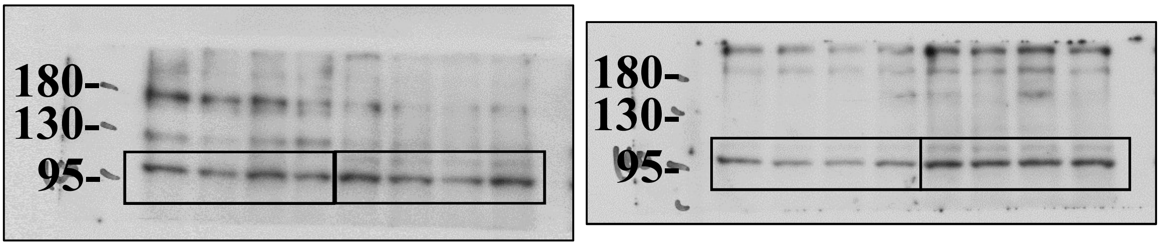

Supplementary Fig 4B BMPR2

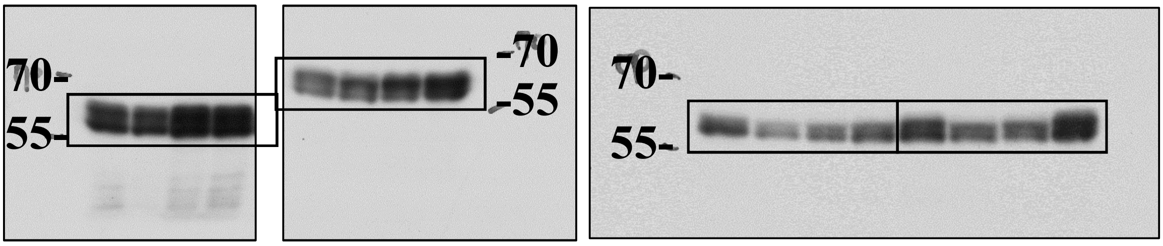

Supplementary Fig 4B pSmad1/5

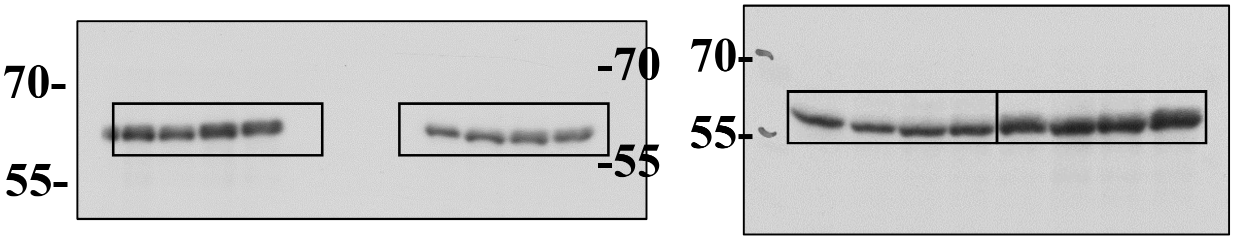

Supplementary Fig 4B Smad1

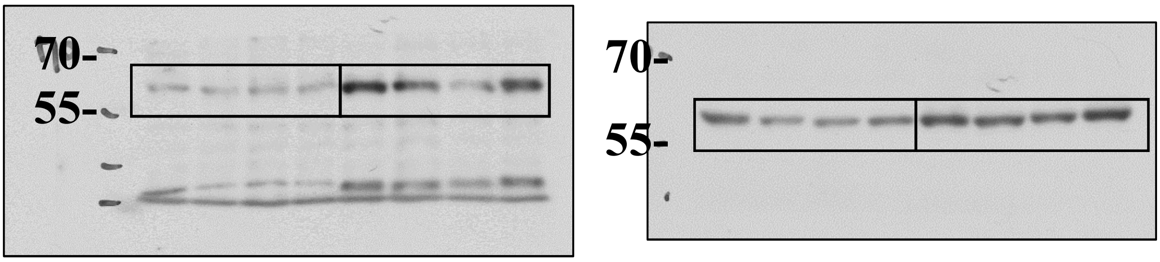

Supplementary Fig 4B Smad5

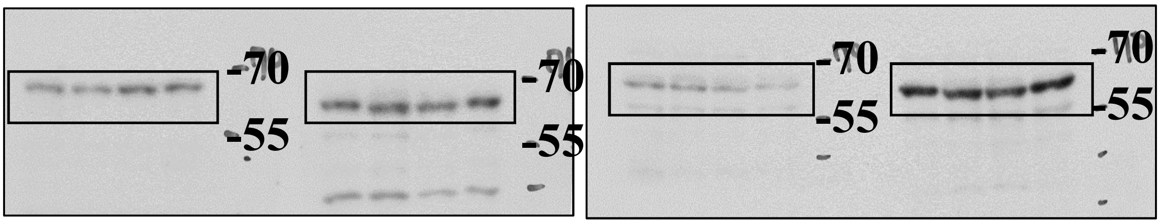

Supplementary Fig 4B Smad4

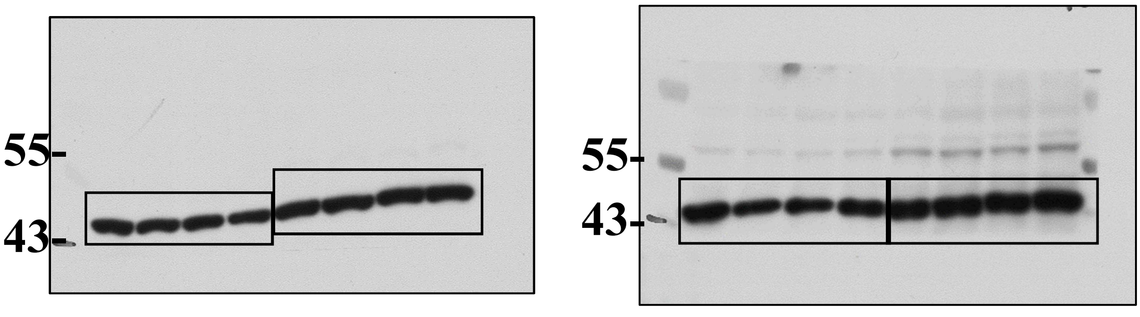

Supplementary Fig 4B β-actin

Supplementary Figure 4C

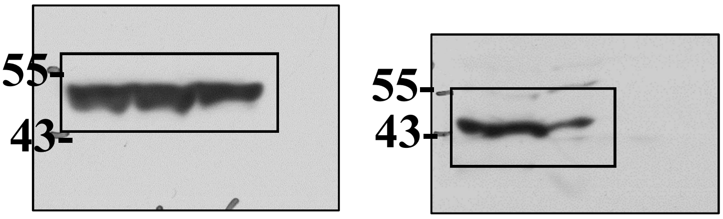

Supplementary Fig 4C BMP2

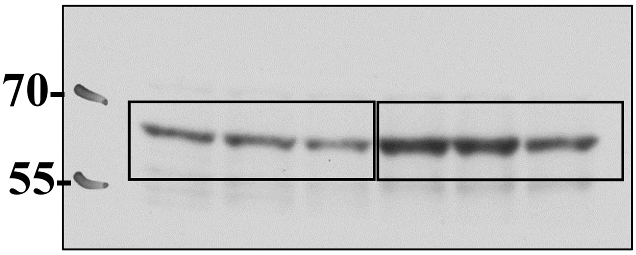

Supplementary Fig 4C Smad4

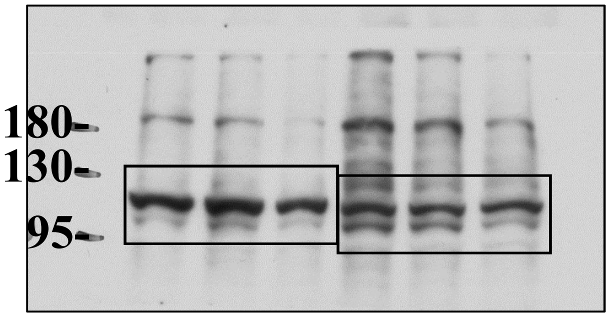

Supplementary Fig 4C BMRP2

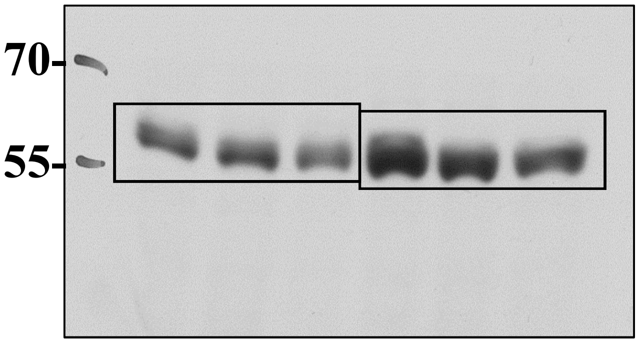

Supplementary Fig 4C Smad5

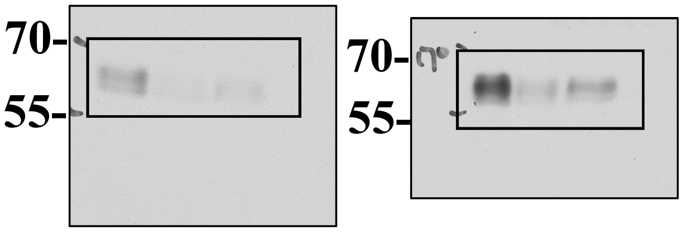

Supplementary Fig 4C pSmad1/5

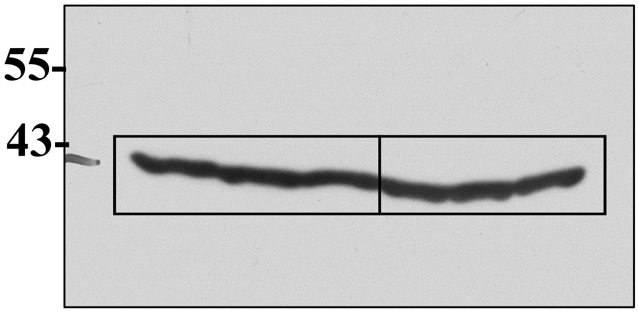

Supplementary Fig 4C β-actin

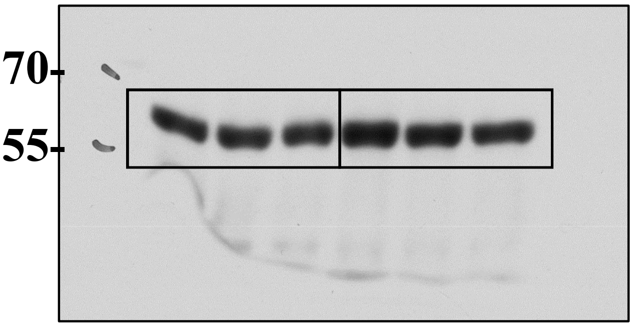

Supplementary Fig 4C Smad1

Supplementary Figure 5A

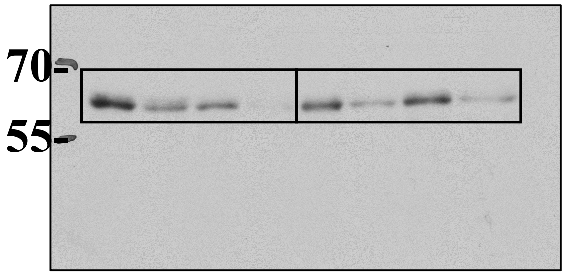

Supplementary Fig 5A Smad1

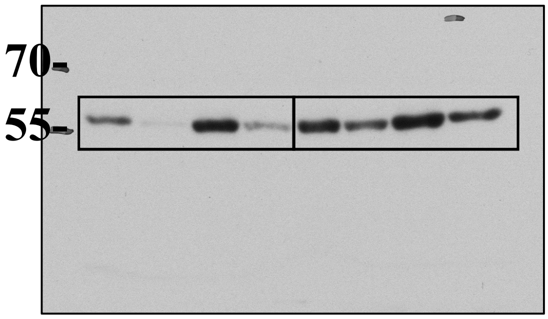

Supplementary Fig 5A Smad5

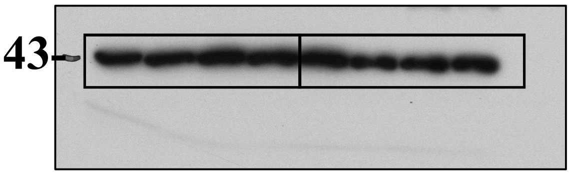

Supplementary Fig 5A β-actin

Supplementary Figure 5B

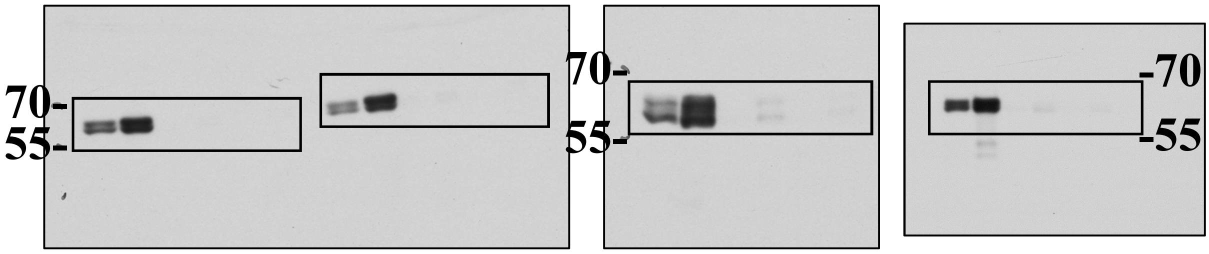

Supplementary Fig 5B pSmad1/5

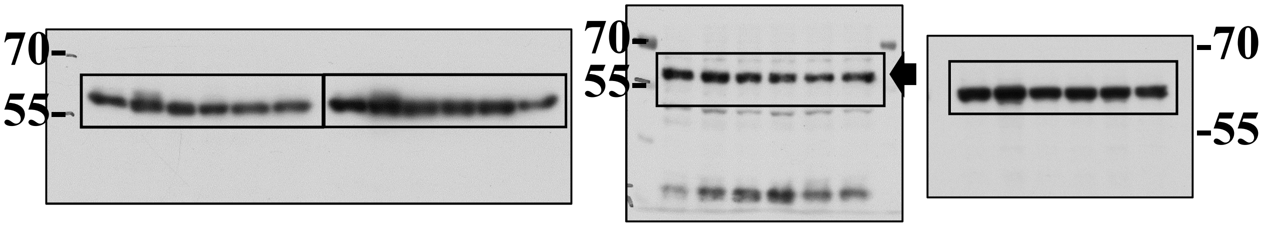

Supplementary Fig 5B Smad5

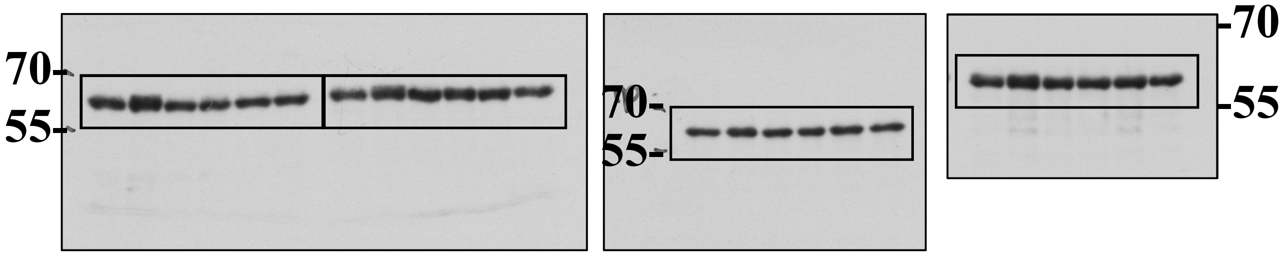

Supplementary Fig 5B Smad1

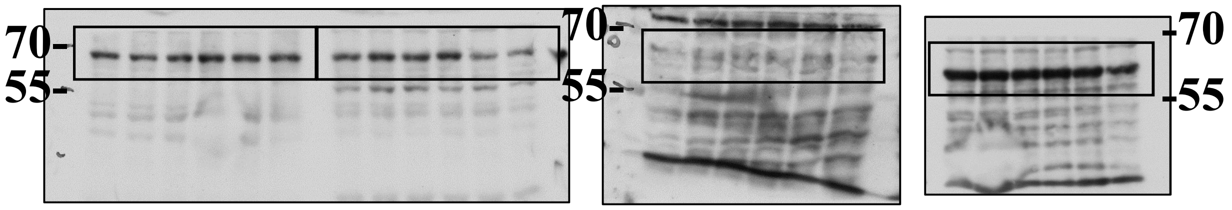

Supplementary Fig 5B Smad4

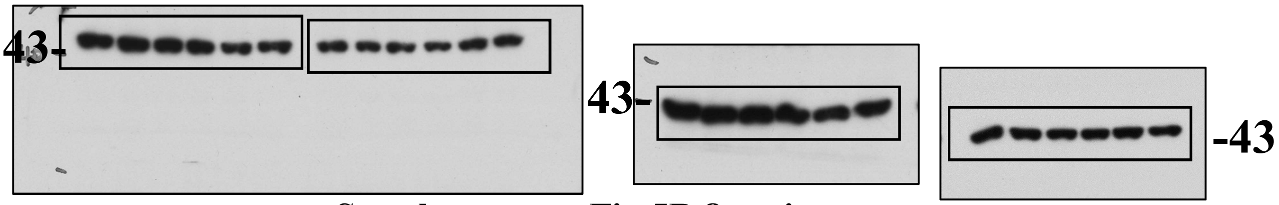

Supplementary Fig 5B β-actin
